# In-depth blood immune profiling of Good syndrome patients

**DOI:** 10.3389/fimmu.2023.1285088

**Published:** 2023-11-15

**Authors:** Alba Torres-Valle, Larraitz Aragon, Susana L. Silva, Cristina Serrano, Miguel Marcos, Josefa Melero, Carolien Bonroy, Pedro Pablo Arenas-Caro, David Monzon Casado, Pedro Mikel Requejo Olaizola, Jana Neirinck, Mattias Hofmans, Sonia de Arriba, María Jara, Carlos Prieto, Ana E. Sousa, Álvaro Prada, Jacques J. M. van Dongen, Martín Pérez-Andrés, Alberto Orfao

**Affiliations:** ^1^ Translational and Clinical Research Program, Centro de investigación del Cáncer (CIC), Instituto de Biología Molecular y Celular del Cáncer (IBMCC), Consejo Superior de Investigaciones Científicas (CSIC) and University of Salamanca (USAL), Salamanca, Spain; ^2^ Cytometry Service, NUCLEUS, Department of Medicine, University of Salamanca, Salamanca, Spain; ^3^ Instituto de Investigación Biomédica de Salamanca (IBSAL), Salamanca, Spain; ^4^ Immunology Department, Donostia University Hospital, Osakidetza Basque Health Service, San Sebastián, Spain; ^5^ Serviço de Imunoalergologia, Centro Hospitalar Universitário Lisboa Norte, Lisbon, Portugal; ^6^ Instituto de Medicina Molecular João Lobo Antunes, Faculdade de Medicina, Universidade de Lisboa, Lisbon, Portugal; ^7^ Servicio de Inmunología, Fundación Jiménez Díaz, Madrid, Spain; ^8^ Department of Internal Medicine, University Hospital of Salamanca, Salamanca, Spain; ^9^ Department of Medicine, University of Salamanca, Salamanca, Spain; ^10^ Servicio de inmunología y genética, Hospital Universitario de Badajoz, Badajoz, Spain; ^11^ Department of Laboratory Medicine, Ghent University Hospital, Ghent, Belgium; ^12^ Department of Diagnostic Sciences, Ghent University, Ghent, Belgium; ^13^ Pediatrics Department, University Hospital of Salamanca, Salamanca, Spain; ^14^ Biomedical Research Networking Centre Consortium of Oncology (CIBERONC), Instituto de Salud Carlos III, Madrid, Spain; ^15^ DNA Sequencing Service (NUCLEUS), University of Salamanca, Salamanca, Spain; ^16^ Bioinformatics service (NUCLEUS), University of Salamanca, Salamanca, Spain; ^17^ Department of Immunology, Leiden University Medical Center, Leiden, Netherlands

**Keywords:** Good syndrome, CVID, hypogammaglobulinemia, combined immunodeficiency, thymoma, immune monitoring, age-related values

## Abstract

**Introduction:**

Good syndrome (GS) is a rare adult-onset immunodeficiency first described in 1954. It is characterized by the coexistence of a thymoma and hypogammaglobulinemia, associated with an increased susceptibility to infections and autoimmunity. The classification and management of GS has been long hampered by the lack of data about the underlying immune alterations, a controversy existing on whether it is a unique diagnostic entity *vs*. a subtype of Common Variable Immune Deficiency (CVID).

**Methods:**

Here, we used high-sensitive flow cytometry to investigate the distribution of up to 70 different immune cell populations in blood of GS patients (n=9) compared to age-matched CVID patients (n=55) and healthy donors (n=61).

**Results:**

All 9 GS patients displayed reduced B-cell counts -down to undetectable levels (<0.1 cells/μL) in 8/9 cases-, together with decreased numbers of total CD4^+^ T-cells, NK-cells, neutrophils, and basophils *vs.* age-matched healthy donors. In contrast, they showed expanded TCRγδ^+^ T-cells (p ≤ 0.05). Except for a deeper B-cell defect, the pattern of immune cell alteration in blood was similar in GS and (age-matched) CVID patients. In depth analysis of CD4^+^ T-cells revealed significantly decreased blood counts of naïve, central memory (CM) and transitional memory (TM) TCD4^+^ cells and their functional compartments of T follicular helper (TFH), regulatory T cells (Tregs), T helper (Th)2, Th17, Th22, Th1/Th17 and Th1/Th2 cells. In addition, GS patients also showed decreased NK-cell, neutrophil, basophil, classical monocyte and of both CD1c^+^ and CD141^+^ myeloid dendritic cell counts in blood, in parallel to an expansion of total and terminal effector TCRγδ^+^ T-cells. Interestingly, those GS patients who developed hypogammaglobulinemia several years after the thymoma presented with an immunological and clinical phenotype which more closely resembled a combined immune humoral and cellular defect, with poorer response to immunoglobulin replacement therapy, as compared to those in whom the thymoma and hypogammaglobulinemia were simultaneously detected.

**Discussion:**

Our findings provide a more accurate definition of the immune cell defects of GS patients and contribute to a better discrimination among GS patients between those with a pure B-cell defect *vs*. those suffering from a combined immunodeficiency with important consequences on the diagnosis and management of the disease.

## Introduction

Good syndrome (GS) is a rare disorder with 530 cases reported until 14^th^ August 2023, which emerges around the fifth decade of life, characterized by coexistence of thymoma and hypogammaglobulinemia (1–15). Similar to other predominantly antibody deficiencies (PAD), like agammaglobulinemia and common variable immunodeficiency (CVID), GS patients suffer from recurrent sinopulmonary infections (2, 3) and less frequently also, gastrointestinal disease (6), some authors suggesting it might represent a severe form of CVID associated with thymoma (16). However, as compared to CVID, GS patients have a higher risk of opportunistic infections, like symptomatic CMV or mucocutaneous candidiasis (17), which are typically associated with a defect of cellular immunity. In addition to infectious complications, autoimmune disorders have also been frequently reported in GS patients, particularly myasthenia gravis, oral lichen planus and pure red cell aplasia. In contrast, organomegalies including hepatomegaly, splenomegaly and lymphadenopathies, are less frequently observed in GS *vs.* CVID (17). Resection of the thymoma has been shown to cure the co-existing myasthenia gravis (18), although it does not seem to be effective in controlling other autoimmune complications of GS, such as oral lichen planus and autoimmune cytopenia (19). In addition, thymectomy does not restore the immunological function of GS patients (20), and immunoglobulin-replacement therapy (IgRT) is required for an improved control of infection. However, IgRT is not equally effective in preventing infections in all GS patients, since up to 20% of GS patients die in the first 5-years after diagnosis (21), mostly due to opportunistic infections associated with T-cell defects (3, 17, 21).

In GS, hypogammaglobulinemia has been associated to very low (usually undetectable) peripheral blood B-cell counts in virtually every patient (2, 3). In addition, a myriad of other T-cell and innate cell defects have been also documented in single case reports (2, 3) and case series from national registries (17, 21, 22). However, part of these latter findings have not been confirmed, and controversial data have been reported about the frequency of TCD4^+^ and TCD8^+^ cell, NK-cell, neutrophil or eosinophil defects in GS patients (2, 3, 17, 21, 22). In part, this is due to the variety and heterogeneity of the methods used (even within the same study) to calculate immune cell counts in blood, and to the lack of age-matched reference controls. The limited robust data available about the immunological profile of GS patients, together with the rarity of the disease, have hampered a comprehensive classification of the disease, which has been included in international guidelines as innate errors of immunity, as a PAD (23, 24) classified within CVID (23) or agammaglobulinemia (24), as combined immunodeficiency (CID) (21), or even as a phenocopy of inborn errors of immunity associated with autoantibodies (25). Of note, only half of all GS patients present at diagnosis (or the onset of the disease) with synchronous thymoma and hypogammaglobulinemia (2, 3), while in 40% of GS patients hypogammaglobulinemia emerges 2-17 years after the diagnosis of thymoma (2, 17, 21) and less frequently (10% of reported cases), hypogammaglobulinemia might preceded in a few years the diagnosis of thymoma (17). Despite such clinical heterogeneity, no studies have been reported so far in which the immunological defects (and other features of the disease) have been compared in these different subgroups of GS patients.

Here, we used for the first time a high-sensitive and validated flow cytometry approach (26–28) for in depth analysis of the distribution of a wide number of relevant immune cell populations in blood of GS patients, compared to a large cohort of age-matched CVID patients and healthy donors.

## Materials and methods

### Patients, controls and samples

A total of 64 adult patients including 9 GS (median age: 66 years; range: 53-79 years; 7 men and 2 women) and 55 CVID patients (median age: 60 years; range: 52-80 years; 28 men and 27 women), diagnosed according to the International Union of Immunological Societies (IUIS) (29) and European Society for Immunodeficiencies (ESID) (30) criteria were studied in parallel to 61 healthy donors (median age: 63 years; range: 52-80 years; 29 men and 32 women). EDTA-anticoagulated peripheral blood (PB) samples were collected at six different centers (University Hospital of Salamanca, Salamanca, Spain; Donostia University Hospital, San Sebastián, Spain; Centro Hospitalar Universitário Lisboa Norte, Lisbon, Portugal; Fundación Jiménez Díaz, Madrid, Spain; Hospital Universitario de Badajoz, Badajoz, Spain; Ghent University Hospital, Ghent, Belgium) and processed within the first 24 hours after collection. Prior to his/her enrolment in the study, each participant gave his/her informed consent to participate in accordance with the guidelines of the local Ethics Committees and the Declaration of Helsinki. The study was approved by the local ethics committees of the participating institutions.

### Flow cytometry screening of primary immunodeficiency of the lymphoid and innate system

Defects in lymphoid and innate cells were investigated using the previously validated primary immunodeficiency orientation tube (PIDOT) method for primary immunodeficiency (PID) screening (26, 31–33) (www.euroflow.org) in blood. Briefly, PB samples were stained with the 8-color, 12-antibody PIDOT using the EuroFlow standard operating procedures (SOP) for staining of surface membrane makers-only available at www.EuroFlow.org, as previously described in detail (26, 31). A total of ≥10^6^ leukocytes stained with the PIDOT antibody combination (**Supplementary Table 1A**) were measured in BD FACSCanto™ II flow cytometers - Becton/Dickinson (BD) Biosciences, San Jose, CA - using the FACSDiva software (BD Biosciences). Automated data analysis was performed with the Infinicyt software - Cytognos SL, Salamanca, Spain -, and the EuroFlow PIDOT database (26, 31) was used for robust and reproducible automated identification of the main populations of leukocytes, including: neutrophils, monocytes, basophils, eosinophils and lymphocytes and their - B-cell, TCRγδ^+^ and TCRαβ^+^ T-cell (e.g., TCD4^+^CD8^-^, TCD4^-^CD8^+^ and TCD4^-^CD8^-^ cells) and NK-cell - subsets.

### Detailed characterization of both blood innate myeloid, T-cell, NK-cell and B-cell populations

Further detailed identification and characterization of up to 8 innate myeloid cell populations, 57 populations of TCD4^+^, TCD8^+^, TCRγδ^+^, and TCD4^-^CD8^-^ TCRαβ^+^ double negative (DN) T-cell and 2 NK-cell populations, and 24 B-cell populations were performed in PB from 8/9 GS patients, 6/55 age-matched CVID patients and 51/61 age-matched healthy donors respectively. Briefly, total monocytes and their classical (cMo), intermediate (iMo) and non-classical (ncMo) monocyte subsets, as well as total dendritic cells (DC) and their CD1c^+^ and CD141^+^ myeloid DC (myDC) and plasmacytoid DC (pDC) were identified as previously described based on the Euroflow immunemonitoring monocyte/dendritic cell (IMM MoDC) tube (28) (**Supplementary Table 1B**). In parallel, CD4^+^ T-cells were split into their maturation associated subsets of naïve, central memory (CM), transitional memory (TM), effector memory (EM) and terminal effector (TE) cells, the later four subsets being further subdivided into their functional subsets based on their surrogated phenotypic profiles -follicular helper T (TFH) cells, regulatory T (Tregs) cells and classical T-helper (Th) cells (Th1, Th2, Th17, Th22, Th1/Th17 and Th1/Th2)- using the EuroFlow 14-color immune monitoring TCD4^+^ (IMM TCD4) tube (27) (**Supplementary Table 1C**). Similarly, CD8^+^, DN-TCRαβ^+^ and TCRγδ^+^ T-cells were grouped into their maturation-associated subsets of naïve, CM, TM, EM, early effector (EE) and TE cells, and total NK cells and their CD56^lo^ and CD56^hi^ NK-cell subsets identified using the EuroFlow 14-colour immunemonitoring cytotoxic (IMM cytotoxic) tube (**Supplementary Table 1D**). When B-cells were detected at a frequency of >0.1 cell/µL, they were analyzed with more detail, including their maturation associated (immature/transitional, naïve and memory B-cells and plasma cells) and IgH isotypic subsets (IgMD, IgG, and IgA) within memory B-cells and plasma cells using the EuroFlow 12-color immunoglobulin heavy chain isotype B-cell (BIgH IMM) tube (34) (**Supplementary Table 1E**). Stained blood samples with the four innate, TCD4, cytotoxic and BIgH IMM tubes was performed based on the EuroFlow SOP for staining of cell surface membrane markers‐only and cell surface membrane plus cytoplasmic markers combined with (innate and BIgH IMM tubes) or without (TCD4 and cytotoxic IMM tubes) the bulk-lysis SOP (www.euroflow.org), as previously described (27, 28, 34–36). For each antibody combination, ≥0.5 x 10^7^ cells (BIgH IMM tube), ≥10^6^ cells (innate IMM tube) and ≥0.5 x 10^6^ cells (TCD4 and cytotoxic IMM tubes) were measured in LSRFortessa X-20 or FACSymphony™ A5 flow cytometers (BD Biosciences), using the FACSDiva software (BD Biosciences). For data analysis the Infinicyt software was used.

### Assessment of soluble plasma immunoglobulin levels

In 80 plasma samples from 9 GS patients, 39/55 CVID patients - median age (range): 62 (52–80) years - and 32/61 healthy donors - median age (range): 74 (52–77) years - soluble immunoglobulin (Ig)G, IgA and IgM levels were quantified using conventional nephelometry (Dimension Vista; Siemens Healthcare, Erlanger, Germany).

### Statistical methods

To assess the statistical significance (set at p-values <0.05) of differences observed between groups, the Mann-Whitney U and the Student T tests for unpaired continuous variables with small-size or a normal distribution were used respectively, while for categorical variables the Chi-square and Fisher exact tests were employed using the SPSS software (version 28.0; IBM, Armonk, NY). For box plot graphical representations, the GraphPad Prism V8 software (GraphPad Software, San Diego, CA) was used. Age-normalized cell counts/µL per patient transformed with a rank order scale were represented in heat maps built with R package ggplot2 (37)⁠. For all immune cell counts in blood, the lower limit of normality (5^th^ percentile) per age-group was defined based on ≥ 20 healthy donors, as previously described (38).

## Results

### Clinical and laboratory features of GS patients

Thymoma was diagnosed simultaneously (time interval <2 months) to hypogammaglobulinemia in 4/9 GS patients, while in the other 5/9 patients hypogammaglobulinemia developed 2-19 years after the thymoma (median of 4 years) (**Table 1**). According to the World Health Organization (WHO) classification (39), half of thymomas were classified as B1 (n=4) or B3 (n=1), 3 patients had AB thymomas, and in 1 patient an A thymoma was diagnosed. Hypogammaglobulinemia was identified based on simultaneously decreased IgG, IgA and IgM serum levels in 67% of cases (including case 9 with 665 mg/dL IgG, close to the lower limit of normality), while in the remaining 3 cases a defect of only two Ig isotypes (IgG/IgA or IgG/IgM) was present. Antibody response to vaccination was absent in 3 patients (cases 2, 7 and 8) and normal in case 9, no data being available for the remaining 5 GS patients.

**Table 1:** Clinical and laboratory characteristics at diagnostic of Good syndrome patients.

	Patient ID								
	Case 1	Case 2	Case 3	Case 4	Case 5	Case 6	Case 7	Case 8	Case 9
**Gender**	M	M	F	M	M	M	F	M	M
**Age at diagnosis (y)**	58	68	43	55	49	48	71	56	60
**Age at inclusion (y)**	63	79	62	69	53	54	73	66	70
Clinical features									
Infections	RTI	RTI	RTI; GI; BC; sepsis	GI	RTI; GI	RTI GI	RTI; UTI; GI; BC	RTI; GI	RTI; GI
Severe Infections	*CMV*	*S. pneumoniae*	*Pseudomonas;* *Pneumocystis;* *Aspergillus*	*H. influenza*	*CMV;* *P. jirovecci*	*Pneumocystis*	*G. lamblia;* *E. coli;* *SARSCoV2*	*Campylobacter*	*H. pylori*
Autoimmunity	No	No	Myasthenia gravis	No	No	No	Myasthenia gravis	No	No
Other Complications	No	No	No	Entheropathy	Bronchiolitis	No	No	No	No
**Thymoma** **(WHO subtype)**	AB	AB	B1	B1	AB	B1	A	B3	B1
**First Thy/H diagnosis**	Thy	Thy	Thy	Thy	Simultaneous	Thy	Simultaneous	Simultaneous	Simultaneous
**Time from Thy/H diagnosis**	4y	2y	19y	5y	≤2m	3y	≤2m	≤2m	≤2m
**IgG (mg/dL)**	38	151	211	212	278	496	529	553	665
**IgA (mg/dL)**	10	52	<5	<5	8	77	<5	58	45
**IgM (mg/dL)**	<21	<7	63	<5	8	13	50	29	19
**Ab response to vaccines#**	NA	Absent (both)	NA	NA	NA	NA	Absent (both)	Absent (both)	Normal
Treatment									
Thymectomy	Yes	Yes	Yes	Yes	Yes	Yes	Yes	Yes	Yes
Chemotherapy	No	No	Yes	No	No	No	No	No	No
Radiotherapy	Yes	No	No	No	No	No	Yes	No	Yes
Ig replacement (initiation)	No	Yes (2y after T)	Yes (19y after T)	Yes (4y after T)	Yes (simultaneous T)	Yes (3y after T)	Yes (simultaneous T)	Yes (3y after T)	No
Outcome after therapy									
Infections	RTI; Sepsis	RTI	RTI; BC	RTI; GI	RTI	RTI	No	RTI; BC	No
Severe infections	*CMV;* *P. aeruginosa*	*S. pneumoniae;* *RSV*	*Pseudomonas;* *Pneumocystis;* *Aspergillus*	*H. influenza;* *SARSCoV2*	*Sistemic CMV;* *SARSCoV2;* *Rhinovirus;* *Enterovirus;* *A. fumigatus*	*-*	*-*	*-*	*-*
Hospital admissions*	> 2	> 2	> 2	> 2	> 2	< 2	< 2	Out-patient	< 2
Autoimmunity (time after therapy)	No	No	Oral lichen planus(16y); Bowen disease(16y); acute diverticulitis(19y); Hypothyroidism(18y)	No	No	No	No	No	No
Other complications (time after therapy)	No	No	Entheropathy (19y); Squamous cell carcinoma (16y)	No	Lymph- adenopathies (1y)	Entheropathy (8y); Splenomegaly (5y)	Gastritis (1y)	No	Prostatic cancer (3y)
**Deaths**	Yes *(CMV;* *P.aeruginosa)*	No	No	No	Yes *(SARSCoV2; Rhinovirus; Enterovirus;* *A. fumigatus)*	No	No	No	No
**Survival** **(y from diagnosis)**	5	11	20	15	10	7	1	18	10

M, male; F, female; y, years; RTI, respiratory tract infections; GI, gastrointestinal infections; BC, bronchiectasis; UTI, urinary tract infection; CMV, Cytomegalovirus; Thy, thymoma; H, hypogammaglobulinemia; m, months; Ig, Immunoglobulin; Ab, antibody; NA, not available; T, thymectomy; RVS, respiratory syncytial virus; Out-patient, antibiotic/antiviral/antifungal prescription treatment. #Ab response to vaccines: Response to both polysaccharide and peptide antigens was tested using pneumococcal polysaccharide and toxoid vaccines respectively; *Hospital admissions related to severe infections.

At diagnosis, all GS patients reported infections. Recurrent respiratory tract infections (RTI) and gastrointestinal (GI) infections were reported in 8 and 7 patients respectively, 2 patients had bronchiectasis, and one had urinary tract infections (UTI). Opportunistic infections were documented in 78% of patients at diagnosis, including opportunistic bacterial (*Pseudomonas, Pneumocystis jirovecci, Campylobacter, Escherichia coli, Helicobacter pylori)*, viral (*Cytomegalovirus -*CMV-) and fungal (*Aspergillus*) infections. In addition, myasthenia gravis was diagnosed in two cases, and enteropathy and bronchiolitis were detected in one case each. No other significant clinical manifestations of disease were documented at diagnosis in the 9 GS patients (**Table 1**).

All 9 GS patients underwent thymectomy shortly after the diagnosis of thymoma. Following the onset of hypogammaglobulinemia, 7/9 patients received IgRT. From the other two patients, one died before starting on IgRT and the other had IgG levels (665 mg/dL) close to the lower limit of normality and did not have severe infections. Cases 5 and 7 received IgRT already at the time of thymectomy, while the remaining patients started on serum Ig therapy a median of 3 years (range 2-19 years) following thymectomy (**Table 1**).

One patient did not report significant infections after IgRT had been established, and IgRT effectively prevented encapsulated bacterial infections and opportunistic infections in 2/4 patients, and 3/6 patients, respectively. However, 5/9 GS patients (56%) required admission to the hospital in >2 occasions due to RTI complications, including 2 cases with bronchiectasis, and 1 case with sepsis. The (more severe) infections consisted of opportunistic infections in all cases, including viral (80% of cases) (respiratory syncytial virus, *CMV*, *Rhinovirus*, SARSCoV2 and *Enterovirus*), bacterial (*Pseudomonas aeruginosa, Pneumocystis*) and fungal (*Aspergillus*) infections. In 2 cases, severe respiratory infections associated with CMV disease were documented 5 and 10 years after the diagnosis of GS. Only 2 cases continued to have encapsulated bacterial infections (*Streptococcus pneumoniae, Haemophylus influenza*). Lymphadenopathies associated with CMV infection were also documented in one case. Myasthenia gravis was effectively treated with thymectomy, although one of the cases subsequently developed several autoimmune complications such as oral lichen planus, complicated with a squamous cell carcinoma, Bowen disease, acute diverticulitis (and enteropathy), and hypothyroidism. In addition, one case developed prostatic cancer, one patient had enteropathy and splenomegaly, and in another one gastritis was documented during follow up.

At the moment of closing this study, overall survival at 5 years from the diagnosis of thymoma and/or hypogammaglobulinemia was of 88% (95% confidence interval - CI -: 68%-100%) (**Supplementary Figure 1**).

### Analysis of classical immune parameters in peripheral blood from GS patients compared to age-matched healthy donors and CVID patients

**Figure 1:**
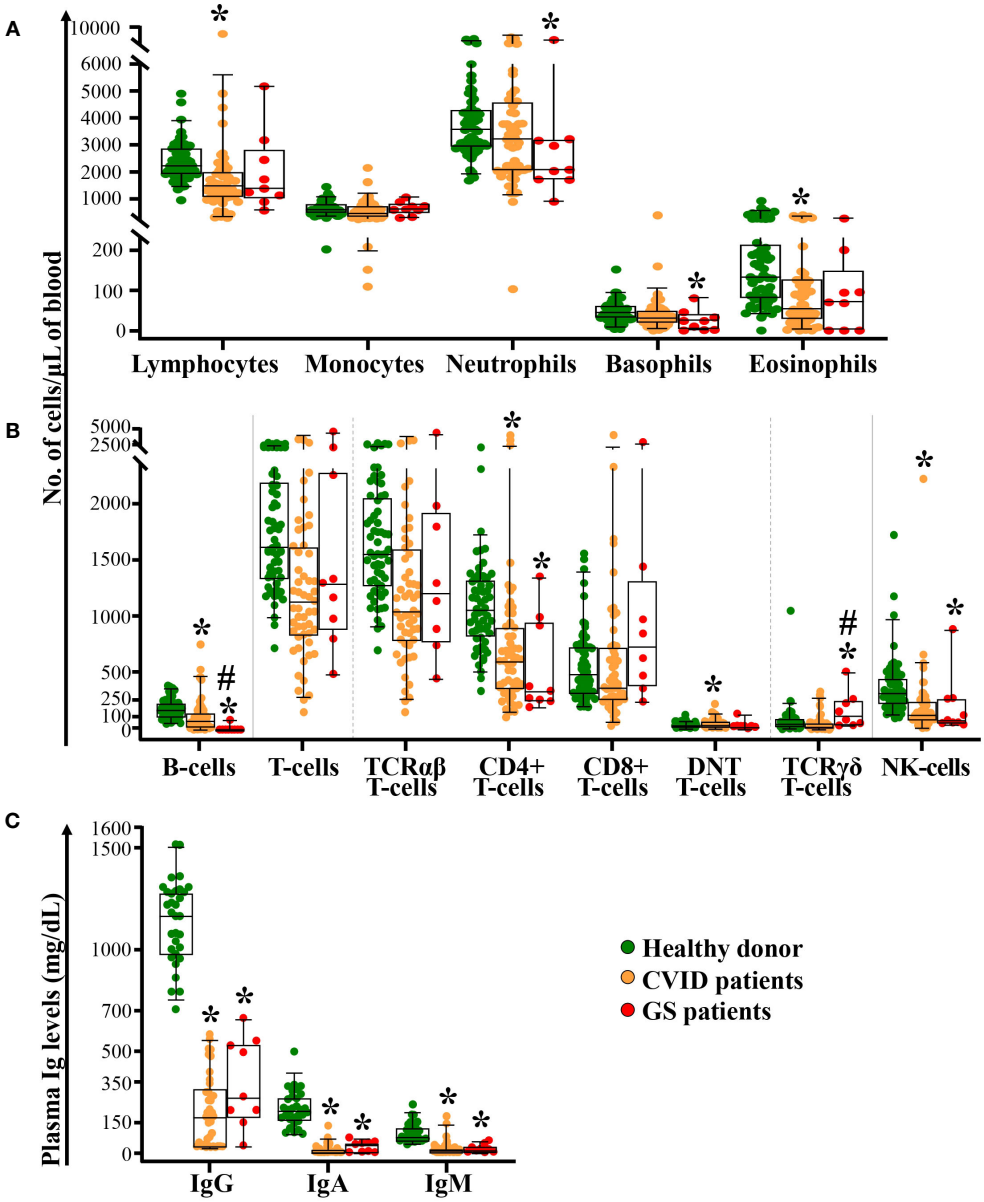
Distribution of different populations of blood leukocytes and lymphocytes, and plasma Ig levels in GS patients compared to both age-matched CVID patients and healthy donors. Absolute counts of monocytes, neutrophils, basophils, eosinophils panel **(A)**, B-cells, T-cells (TCRαβ^+^ [including TCD4^+^, TCD8^+^ and DNT cells] and TCRγδ^+^) and NK-cells panel **(B)** in blood of GS patients (n=9) compared to age-matched CVID patients (n=55) and healthy donors (n=61), are shown in panels **(A, B)** In panel **(C)**, the levels of soluble IgG, IgA and IgM in plasma of GS patients (n=9) compared to age-matched CVID patients (n=39) and healthy donors (n=32), are displayed. Notched boxes extend from the 25^th^ to the 75^th^ percentile values, the lines in the middle and vertical lines correspond to median values and the 5^th^ and 95^th^ percentiles, respectively; individual cases are represented as green dots (healthy donors), orange dots (CVID) and red dots (GS). CVID, common variable immunodeficiency; GS, Good syndrome; TCR, T-cell receptor; DNT, double negative T-cells (CD4^-^CD8^-^ TCRγδ^-^); NK, natural killer; n, number of cases; Ig, immunoglobulin. *p-value ≤ 0.05 *vs.* healthy donors. #p-value ≤ 0.05 *vs.* CVID.

All GS patients consistently presented hypogammaglobulinemia with statistically significantly (p<0.001) decreased IgG, IgA and IgM levels compared to age-matched healthy donors (**Figure 1**). In 8/9 and in 1/9 GS patients, hypogammaglobulinemia was associated with complete lack (<0.1 cells/µL) or strongly decreased (89 cells/μL) circulating B-cells in blood, respectively (p<0.001 *vs.* age-matched healthy donors) (**Figure 1**). Additionally, important numerical alterations involving other immune cells populations were also found as compared to age-matched healthy donors. Those included significantly (p ≤ 0.01) decreased counts of neutrophils, basophils, total CD4^+^ T-cells and NK-cells, together with significantly expanded TCRγδ^+^ T-cells (p = 0.04) (**Figure 1**). Total lymphocyte and eosinophil counts in blood also tended to be decreased, although differences did not reach statistical significance (p=0.06).

A similar pattern of immune cell alterations was found in a subgroup of age-matched CVID patients (*vs.* healthy donors), including statistically significantly decreased serum IgG, IgA and IgM levels, and reduced counts of B-cells, total lymphocytes, total CD4^+^ T-cells, NK-cells and eosinophils, in association with a tendency towards higher TCRγδ^+^ T-cell counts (p=0.07) and monocytopenia (p=0.051) (**Figure 1**). Differences between GS and age-matched CVID patients were restricted to significantly lower B-cell (p<0.001) and higher TCRγδ^+^ T-cell counts (p=0.003) in the former group.

### In depth detailed dissection of T-cell, NK-cell, monocyte, DC and B-cell populations in blood of GS patients *vs*. age-matched CVID patients and healthy donors

**Figure 2:**
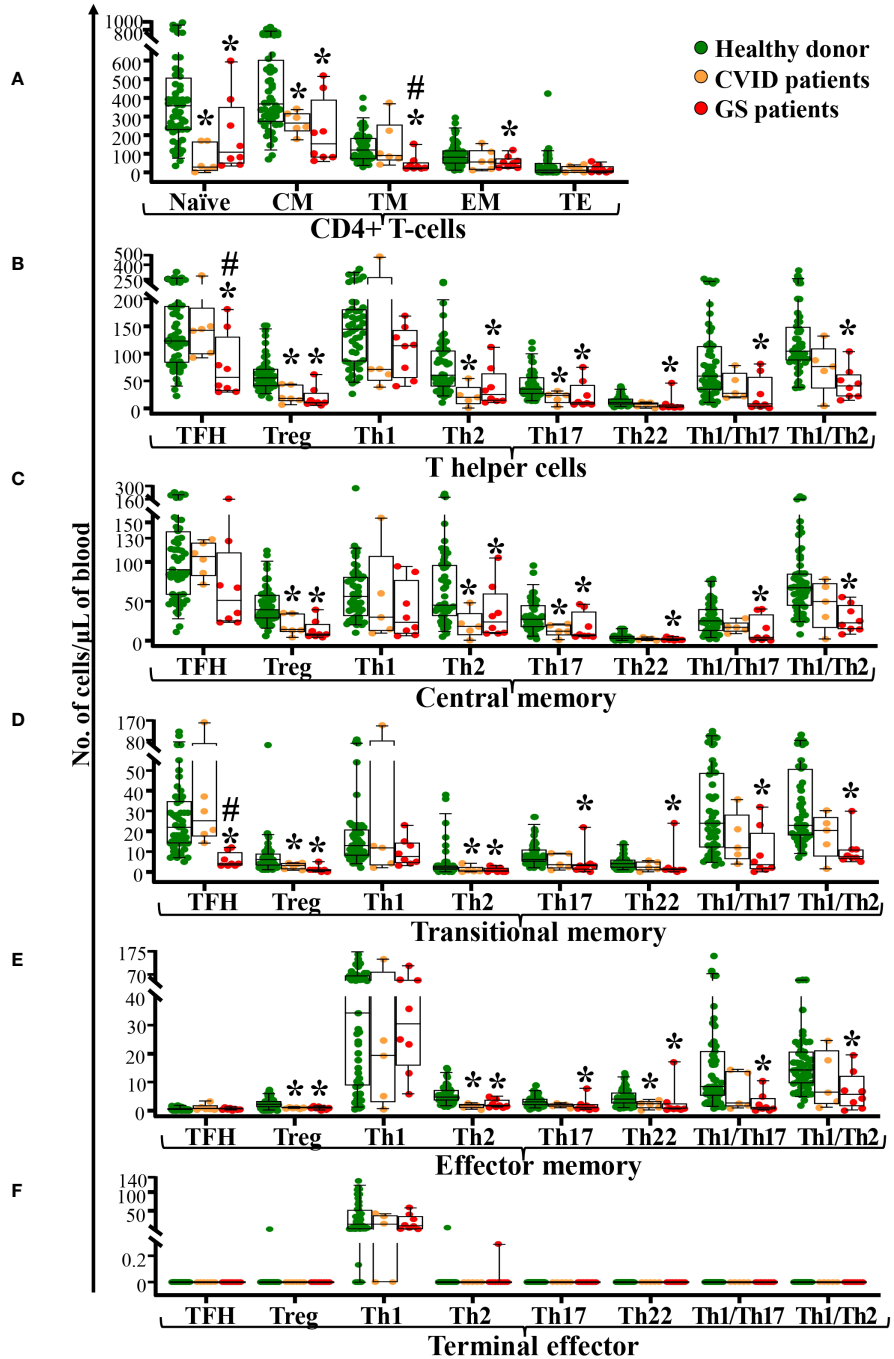
Distribution of maturation-associated subsets and functional populations of CD4^+^ T-cells in blood of GS patients (n=8) compared to age-matched CVID patients (n=6) and healthy donors (n=51). Absolute counts of maturation-associated subsets of CD4^+^ T-cells (naïve, central memory -CM-, transitional memory -TM-, effector memory -EM-, and terminal effector -TE- cells) panel **(A)** and functional subsets (TFH, Treg, Th1, Th2, Th17, Th22, Th1/Th17 and Th1/Th2) panel **(B)** of central memory CD4^+^ T-cells panel **(C)**, transitional memory CD4+ T-cells panel **(D)**, effector memory CD4^+^ T-cells panel **(E)** and terminal effector CD4+ T-cells panel **(F)** in blood of GS patients *vs.* age-matched healthy donors, are shown. Notched boxes extend from the 25^th^ to the 75^th^ percentile values, while the line in the middle and vertical lines correspond to median values and the 5^th^ and 95^th^ percentiles, respectively; individual cases are represented as green dots (healthy donors), orange dots (CVID) and red dots (GS). CVID, common variable immunodeficiency; GS, Good syndrome; n, number of cases; TFH, follicular helper T-cells; Treg, regulatory T-cells; Th, T helper cells. *p-value ≤ 0.05 *vs.* healthy donors. #p-value ≤ 0.05 *vs.* CVID.

Detailed dissection of CD4^+^ T-cells (16, 23) by maturation stage revealed that the total CD4^+^ T-cell defect observed in GS patients (*vs.* age-matched healthy donors) was due to a significant decrease in naïve (p=0.03) CD4^+^ T-cells, in addition to specifically reduced counts of Tregs, Th2, Th17, Th22, Th1/Th17 and Th1/Th2 cells within the CM (p<0.03), TM (p<0.04) and EM (p<0.01) compartments of CD4^+^ T-cells (**Figure 2**). In addition, reduced CM (p=0.06) and particularly, TM (p<0.001) TFH cell counts were also observed, in association with virtually undetectable but normal, EM TFH cell counts. In turn, Th1 CM cells tended (p=0.06) to be lower among GS patients *vs.* healthy donors, whereas no differences were observed between GS patients and age-matched controls as regards the Th1 TM, Th1 EM, and Th1 TE compartments (**Figure 2**). These differences translated into significantly decreased numbers in blood of GS patients of total CM, TM and EM CD4^+^ T-cells (p<0.05), total TFH, Tregs, Th2, Th17, Th22, Th1/Th17 and Th1/Th2 CD4^+^ T-lymphocytes (p<0.02) (**Figure 2**). Although CVID patients also showed decreased counts of several TCD4^+^ subsets (**Figure 2**), total TFH and TM TFH were significantly reduced in GS *vs.* age-matched CVID patients (p ≤ 0.05).

**Figure 3:**
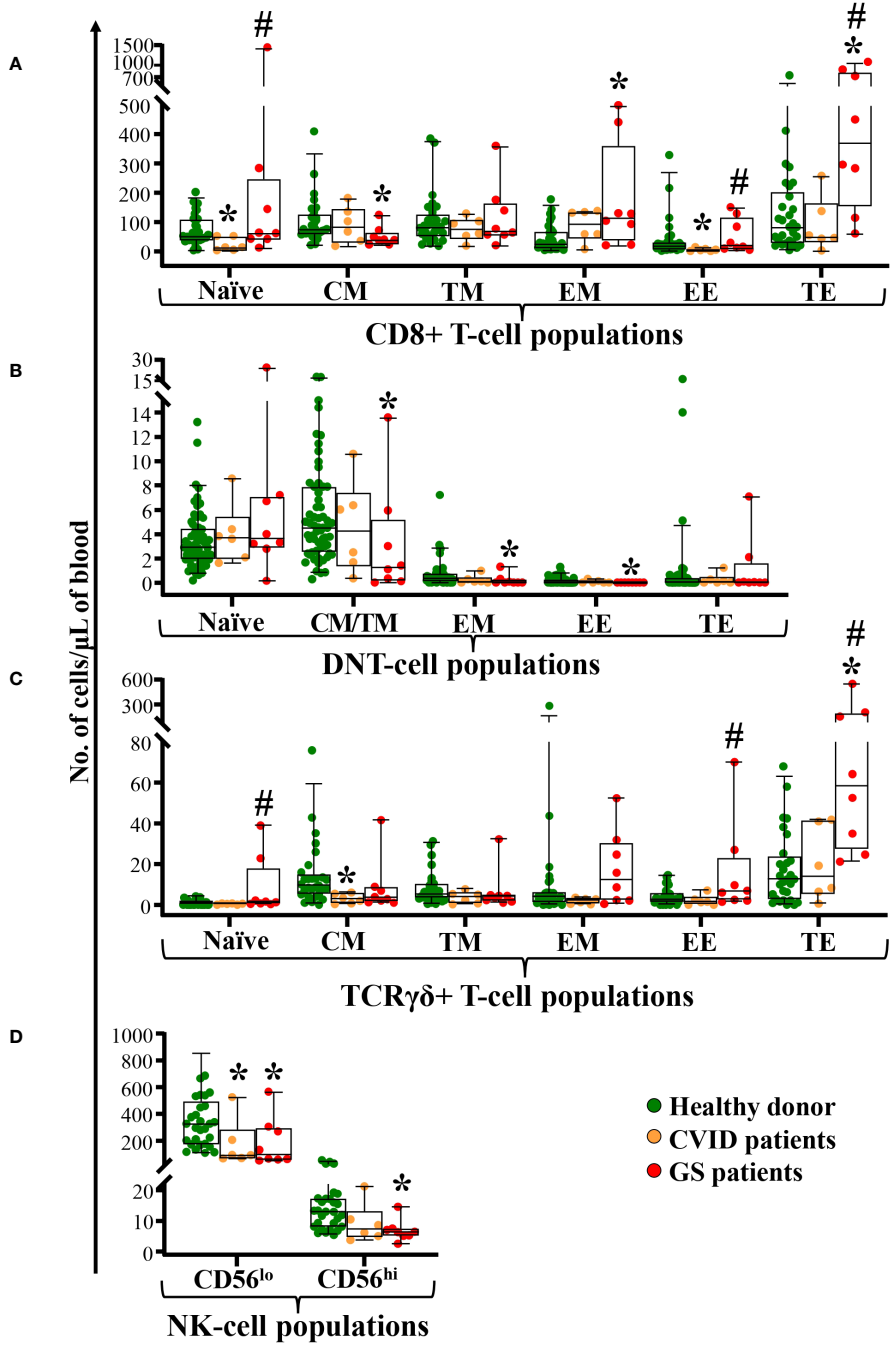
Distribution of different maturation-associated populations of cytotoxic T-cells and NK-cells in blood of GS patients (n=8) compared to age-matched matched CVID patients (n=6) and healthy donors (n=51). Absolute counts of different subsets of blood CD8^+^ T-cells, DN-TCRαβ^+^ T-cells, and TCRγδ^+^ T-cells defined according to their maturation stage (naïve, central memory -CM-, transitional memory -TM-, effector memory -EM-, early effector -EE-, and terminal effector -TE-) are shown in panels **(A–C)** respectively. In panel **(D)** NK-cell subsets defined by the levels of CD56 expression in peripheral blood from patients with GS and age-matched healthy donors. Notched boxes extend from the 25^th^ to the 75^th^ percentile values, whereas the line in the middle and vertical lines correspond to median values and the 5^th^ and 95^th^ percentiles, respectively; individual cases are represented as green dots (healthy donors), orange dots (CVID) and red dots (GS). CVID, common variable immunodeficiency; GS, Good syndrome; n, number of cases; DNT, double negative T-cells (CD4^-^CD8^-^ TCRγδ^-^); TCR, T-cell receptor; NK, natural killer. *p-value ≤ 0.05 *vs.* healthy donors. #p-value ≤ 0.05 *vs.* CVID.

Naïve TCD8^+^, TCRγδ^+^ and DN-TCRαβ^+^ T-cells were within the normal range in GS patients (**Figures 3A–C**). In contrast, significantly reduced CM CD8^+^ T-cell counts (p=0.007) and significantly increased EM and TE CD8^+^ T-cell counts (p ≤ 0.01) were observed in GS patients compared to age-matched healthy donors (**Figure 3A**). Within TCRγδ^+^ T-cells, a tendency towards lower CM (p=0.06) cell counts was observed together with significantly expanded TE TCRγδ^+^ T-cells (p<0.001) (**Figure 3B**). In contrast, almost all maturation subsets of DN-TCRαβ^+^ T-cells (EM, TM, EM and EE) evaluated were reduced (p ≤ 0.02 *vs.* healthy donors), whereas TE DN-TCRαβ^+^ T-cells were within the normal range (**Figure 3C**). This was associated with reduced counts of total NK-cells in GS patients due to significantly decreased CD56^lo^ and CD56^hi^ NK-cells (p ≤ 0.02 *vs.* healthy donors) (**Figure 3D**). As compared to age-matched CVID patients, significantly higher counts of both naïve CD8^+^ and TCRγδ^+^ T-cells, in addition to both TCD8^+^ and TCRγδ^+^ T-cells TE/EE (p<0.05) were observed in GS patients (**Figure 3**).

**Figure 4:**
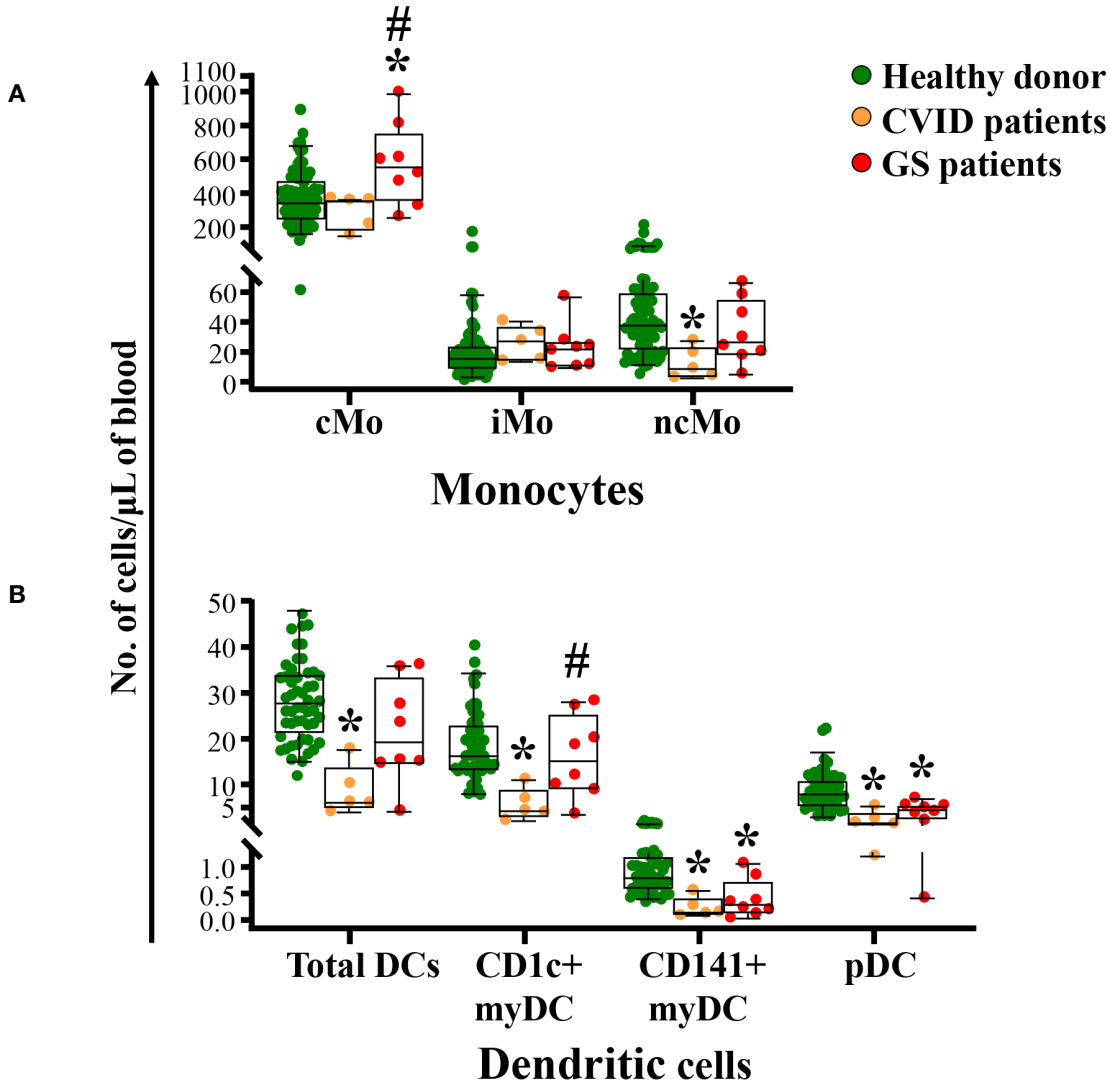
Distribution of different populations of monocytes and dendritic cells in blood of GS patients (n=8) compared to age-matched matched CVID patients (n=6) and healthy donors (n=51). Absolute counts of the major subsets of monocytes (cMo, iMo and ncMo) panel **(A)** and both total DC and their subsets of total myDC, CD1c^+^ myDC, CD141^+^ myDC and pDC panel **(B)** in blood of GS patients *vs.* age-matched healthy donors, are shown. Notched boxes extend from the 25^th^ to the 75^th^ percentile values, while the line in the middle and vertical lines correspond to median values and the 5^th^ and 95^th^ percentiles, respectively; individual cases are represented as green dots (healthy donors), orange dots (CVID) and red dots (GS). CVID, common variable immunodeficiency; GS, Good syndrome; n, number of cases; Mo, monocytes; DC, dendritic cells; cMo, classical monocytes; iMo, intermediate monocytes; ncMo, non-classical monocytes; myDC, myeloid dendritic cells; pDC, plasmacytoid dendritic cells. *p-value ≤ 0.05 *vs.* healthy donors. #p-value ≤ 0.05 *vs.* CVID.

Although no differences were observed between GS patients and age-matched healthy donors in total monocyte counts in blood, detailed dissection of this cell populations showed increased counts of cMo in GS patients when compared to age-matched healthy donors (p=0.01) and CVID patients (p=0.04) (**Figure 4A**). In contrast, GS patients showed decreased numbers of both CD141^+^ myDC and pDC in blood (**Figure 4B**) compared to age-matched healthy donors (p ≤ 0.002) while CD1c^+^ myDC were preserved and higher than in age-matched CVID patients (p=0.04).

In only 1/9 GS patients (Case 9) B-cells were detected at a frequency at ≥0.1 cell/µL. In this patient, the circulating plasma cell count was close to the lower values observed in age-matched healthy controls (0.3 *vs.* 0.3-11 cells/µL), including low counts of IgM^+^ (0.03 *vs.* 0.02-1.0 cells/µL), IgG^+^ (0.08 *vs.* 0.06-2.8 cells/µL) and IgA^+^ (0.2 *vs*. 0.2-6.6 cells/µL) plasma cells, whereas the number of immature/transitional, naïve B-cells and IgMD^+^, IgG^+^ and IgA^+^ memory B-cells from this single case were within the p10-p90 range observe in age-matched healthy donors(data not shown).

### Clinical features and immune profile of GS patients stratified by the time lapse between the diagnosis of thymoma and the onset of hypogammaglobulinemia

**Figure 5:**
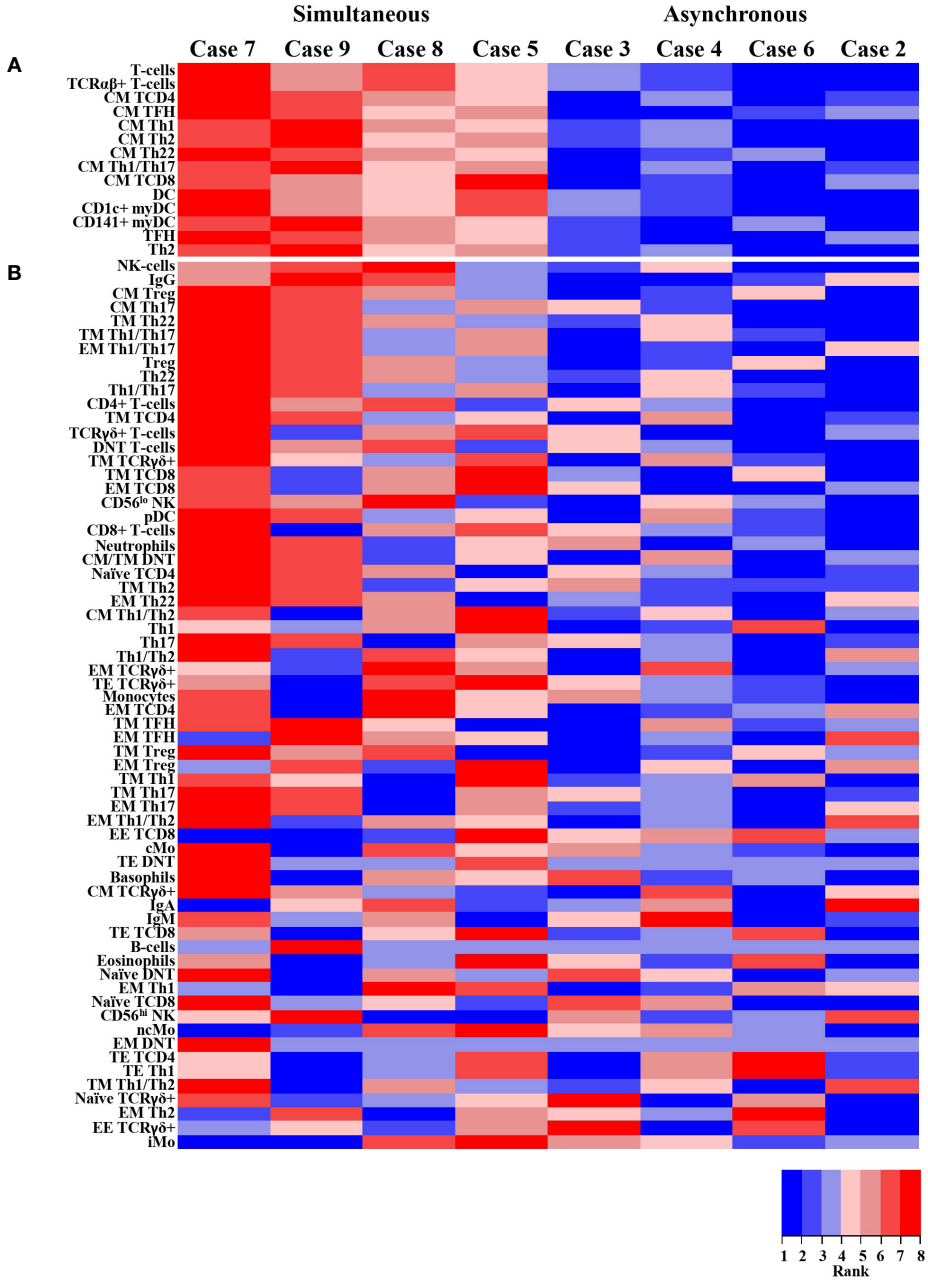
Heat map graphical representation of the blood immune cell profile and Ig serum levels of GS patients (n=8) stratified according to the time lapse between the diagnosis of thymoma and the onset of hypogammaglobulinemia. The heat map representation is based on the absolute counts of the different populations of immune cells identified in blood (75 parameters) and Ig serum levels (IgG, IgA and IgM) normalized by the LLN found among healthy donors for the corresponding age group (rows) versus individual GS patients (columns) grouped according to the time lapse between the diagnosis of thymoma and the onset of hypogammaglobulinemia (i.e., simultaneous *vs.* asynchronous diagnosis of thymoma and hypogammaglobulinemia). In the upper part parameters with a p-value ≤ 0.05 for the comparison between GS patients with simultaneous *vs.* asynchronous diagnosis of thymoma and hypogammaglobulinemia is shown panel **(A)**, while those parameters that did not show statistically significant differences between both patient groups are represented in the lower part of the graph panel **(B)**. The data were transformed with a rank order (colored) scale, where red represents increased levels and blue corresponds to decreased levels. EE DNT cells and all functional subsets of TE compartment (except TE Th1) are not represented because they were undetectable in almost all GS patients. GS, Good syndrome; n, number of cases; Th, T helper; H, hypogammaglobulinemia; Ig, immunoglobulin; LLN, lower limit of normality; TCR, T-cell receptor; DNT, double negative (CD4^-^CD8^-^ TCRαβ^+^) T-cells; TFH, follicular helper T-cells; Treg, regulatory T-cells; CM, central memory; TM, transitional memory; EM, effector memory; EE, early effector; TE, terminal effector; NK, natural killer; Mo, monocytes; cMo, classical monocytes; iMo, intermediate monocytes; ncMo, non-classical monocytes; DC, dendritic cells; myDC, myeloid dendritic cells; pDC, plasmacytoid dendritic cells.

From a clinical point of view, GS patients were divided in two groups according to the time lapse observed between the diagnosis of thymoma and the onset of hypogammaglobulinemia (**Table 1**), depending on whether the diagnosis of both conditions occurred simultaneously (<2 months) or asynchronously (2-19 years). Of note, clearly distinct patterns of alteration of specific T-cell and innate cell populations were observed between these two groups (**Figure 5**). Thus, compared to patients with a simultaneous diagnosis of thymoma and hypogammaglobulinemia, GS cases with an asynchronous diagnosis of both disease conditions, showed statistically significantly lower counts of total T-cells, TCRαβ^+^ T-cells, total CM TCD4^+^ cells and CM TFH, Th1, Th2, Th22 and Th1/Th17 CD4^+^ T-cells, total TFH CD4^+^ T-cells, Th2 CD4^+^ T-cells, CM CD8^+^ T-cells, total DCs and both CD1c^+^ myDC and CD141^+^ myDC (p=0.03). In addition, patients with an asynchronous diagnosis also tended (p=0.06) to have lower serum IgG levels and lower counts of NK cells, CM Treg, CM Th17, TM Th22, TM and EM Th1/Th17, Treg CD4+ T-cells, Th22 CD4^+^ T-cells and Th1/Th117 CD4^+^ T-cells in blood (**Figure 5**).

Once IgRT had been established, asynchronous GS patients showed a higher frequency of opportunistic infections compared to GS patients with a simultaneous diagnosis of thymoma and hypogammaglobulinemia (100% *vs.* 25%, respectively; p=0.05) (**Supplementary Table 2**). The increased frequency of opportunistic infections was associated with a lower 5-year overall survival rate of 80% *vs.* 100%, in asynchronous *vs.* simultaneous GS patients, although it did not reach statistical significance (**Supplementary Figure 1**).

## Discussion

Our understanding of the pathophysiologic mechanisms that lead to the unique association between thymoma and immunodeficiency has not advanced significantly for decades, since the first description of GS in the 50’s (1). This is due to the fact that in contrast to other PAD (40–45), only a few immunological studies have been reported which are focused on GS patients, most available data being restricted to serum Ig levels and total TCD4^+^, TCD8^+^ cell and B-cell counts (2, 3, 17, 21, 22). Additionally, the interpretation of the above immunological findings has further been hampered by the lack of robust age-matched reference ranges. Although GS has been suggested to be a subtype of CVID with an associated thymoma, no previous studies have compared in detail the underlying defects in both groups of patients. Thus, current knowledge about the underlying immune defects in GS patients is mostly based on summary reports of cases from national/international PID registries (17, 21, 22) and reviews of the pre-existing literature (2, 3).

Here, we investigated in depth for the first time, the distribution of different immune cell compartments in blood of GS patients based on standardized and previously validated high-sensitive flow cytometry methods (26, 33), and compared it to a series of age-matched healthy donors and CVID patients. Overall, our results confirm that in addition to (virtually) undetectable B-cells and decreased serum Ig levels (1), GS patients also have decreased counts of circulating CD4^+^ T-cells (2, 3, 46, 47), NK-cells (2, 22, 46, 48, 49), basophils (50) and neutrophils (2, 3, 46), associated with a significant expansion in blood of TCRγδ^+^ T-cells (51). Although controversial data about these subsets have been previously reported in the literature (2, 17, 52), this might be due to the lack of age-matched controls and the broad range of (heterogeneous) methods used in the multiple centers reporting on GS patients. In this regard also, despite increased CD8^+^ T-cell counts in blood have been found in some studies (2, 3), in our cohort differences which did not reach statistical significance with around half of our GS patients displaying CD8^+^ T-cell counts within normal range. Moreover, although GS patients with undetectable eosinophils in blood have been reported in some studies (2, 50), in our cohort only a trend (without statistical significance) towards decreased eosinophil counts was observed in blood of GS patients when compared to healthy donors, with undetectable or strongly reduced (0.3 cells/µL) eosinophil counts being found in only 2/9 and 1/9 GS patients, respectively.

Despite several authors have previously suggested that GS might be a form of CVID with thymoma (22), in only one study the blood immune cell profile of these two diseases has been compared so far, based on younger CVID (*vs.* GS) patients and focused on CD4^+^ T-cells which were found to be more prominently decreased in GS *vs.* CVID (17). In contrast with these data, here we did not observe statistically significant differences between GS and age-matched CVID patients for most immune cell populations evaluated, except for significantly lower B-cell counts (undetectable using conventional cytometry methods at levels of <0.1 cell/µL in 8/9 GS patients), together with greater TCRγδ^+^ T-cell counts, found among GS *vs.* age-matched CVID patients. However, lack of detectable B-cells in adults is not exclusive of GS, and immunophenotypic studies of large cohorts of CVID patients have revealed that up to 15% of adult CVID patients also display a severe B-cell defect (17, 38, 53). Interestingly, the clinical phenotype of these B-negative CVID patients was closer to that of GS cases, both being associated with a higher rate of opportunistic infections in comparison with other PAD cases (17, 38, 53), although CVID B-negative patients display a greater frequency of splenomegaly than that observed in GS cases in the absence of thymoma.

Detailed immunophenotypic analysis of the TCD4^+^ and TCD8^+^ cell populations have shown a wide range of abnormalities in CVID, particularly among those patients that present with more severe clinical complications of the disease (40–45, 54–58). Thus, in addition to the B-cell defect, CVID patients might also display a) an exhausted thymic output (41) associated with significantly reduced naïve TCD4^+^ and naïve TCD8^+^ cell counts (17, 41, 42) and, b) Th1-mediated inflammatory processes (40, 59) associated with an increased frequency of circulating Th1/TFH CD4^+^ T-cells (44, 45, 60) and both EM and TE TCD8^+^ and TCRγδ^+^ T-cells (42, 55, 58, 61), in parallel to reduced Treg, Th2, Th17 and CM CD4^+^ T-cells (42, 61). Although evidence of T-cell defects, either as cutaneous anergy, impaired response to T-cell mitogens, or decreased *in vitro* IL-2 production, has been observed in several single-case reports (2, 62) in-depth investigations of blood T-cell populations in GS patients have not been carried out in a systematic way. In this regard, our results suggest that, despite the pattern of alterations in the distribution of the major lymphoid and innate cell populations (and the serum Ig levels) found in GS patients is partially shared with CVID, the former patients also showed unique immune cell profiles in blood, which are significantly different from those of CVID, and that might contribute to explain the existence of distinct pathogenic mechanisms in GS and CVID. Thus, while GS patients displayed decreased counts of CD4^+^ T-cells in blood, the thymic output defect did not affect significantly the naïve TCD8^+^ and TCRγδ^+^ T-cell counts in blood, the number of these cells being significantly higher in GS patients compared to age-matched CVID patients. In turn, a wider defect in the memory CD4^+^ T-cell maturation compartment was observed in GS, as compared to CVID patients (41, 42), which involved not only the (reduced) Th2, Th17 and Treg cells, but also the CM/TM, Th22, Th1/Th2, Th1/Th17 and TFH cells, whose counts were found to be also significantly reduced in blood of GS (*vs.* age-matched healthy donors) but not of CVID patients (42, 44, 45, 60, 61). This translates into a higher relative frequency of Th1 cells within the TCD4^+^ compartment of GS patients. Interestingly, association between reduced TFH cell counts observed in GS patients, could not be confirmed in a limited cohort of age-matched CVID patients, suggesting that this defect might be specifically associated with the thymoma. In addition to the classical TCD4^+^ helper cells, also DN-TCRαβ^+^ T-cells, a cell population that comprises both T helper and Treg cells (63), was defective in GS patients. In contrast, an expansion in both absolute counts and relative numbers of both EM and TE TCD8^+^ cells was observed in GS patients as compared to age-matched healthy donors and CVID patients, in line with previous findings in two GS single-case reports (20, 64), which might be due to prolonged antigen stimulation associated with e.g., chronic infections. In fact, as for CVID (53), the largest expansions of EM/TE TCD8^+^ cells observed in blood of our cohort were found among GS patients who had systemic (symptomatic) CMV infection. Interestingly, the above reported naïve/memory TCD4^+^ defect observed in GS patients was not isolated. In contrast, it was associated with significantly lower counts in blood of both CD141^+^ myDC and pDC. Although decreased counts of myDC and pDC have been also reported in CVID patients (65, 66) and in a GS single-case report (20), their potential association with specific T-cell defects has not been previously established. Interestingly, in our cohort, there was a significant correlation between the CD141^+^ myDC counts and both the naïve TCD4^+^ and CM/TM TCD4^+^ cell numbers in blood (**Supplementary Figure 2**), suggesting that a reduction in the number of circulating DC in blood might exert a negative impact in the priming and maintenance of naïve and long-lived memory CD4^+^ T-cells, respectively (67, 68). Interestingly, DC and T-cell defects could be associated with the previously reported presence of anti-cytokine autoantibodies, including IL-17A, IL-22. IL-17F, IL-12p70 or type I IFN, reported in thymoma patients (69). However, we also observed a wide spectrum of lymphoid and innate immune cell defects among our GS patients, suggesting that (70), there might be different PID-related pathogenic mechanisms leading to thymoma with hypogammaglobulinemia.

In only half of our GS patients, hypogammaglobulinemia was present at the moment the diagnosis of thymoma had been established. In the other half of the patients, the antibody defect emerged subsequently, between 2 and 18 years after thymectomy. Despite such different immune profiles have been systematically observed in previously reported GS cohorts, no studies have questioned so far, whether these two groups of patients are associated with different immune cell profiles in blood and potentially also, with distinct pathogenic mechanisms. Overall, our data showed that, while a predominantly humoral defect was already observed at diagnosis in patients with simultaneous onset of thymoma and hypogammaglobulinemia, traces of a combined immunodeficiency were already observed also among those patients that only developed hypogammaglobulinemia several years after thymectomy. Thus, while in the former GS patient group immune alterations were restricted to reduced serum Ig levels and lack of detectable B-cells, those patients who presented with a delayed onset of hypogammaglobulinemia specifically displayed reduced counts of CM TCD4^+^ cells (including TFH, Th1, Th2, Th22 and Th1/Th17 cells), total TFH, total Th2, CM TCD8^+^ cells and both total DC, and their CD1c^+^ myDC and CD141^+^ myDCs subsets. These data support previous hypothesis raised already more than 40 years ago (71) suggesting that GS might emerge from two different forms of immunodeficiency, either a PAD-like disease or a combined immunodeficiency (CID)-like disorder. For several decades, the pathophysiological classification of GS has remained elusive. Thus, the International Union of Immunological Societies (IUIS) has successively suggested that GS could be classified either as a “PID associated/secondary to other diseases”, as CVID or as CID (1999) (23), as a form of agammaglobulinemia (2005) (24) or more recently, as a phenocopy of PID induced by autoantibodies (2017) (25), without providing any pathophysiological evidences for such classifications. Despite previous evidence exists which support a T-cell functional defect in GS, (71, 72) no biomarkers that might help to discriminate PAD-like from CID-like forms of GS such as those described here for the first time, had been previously identified. In this regard, our results also suggest that IgRT would only be more effective in controlling opportunistic infections in GS patients with a PAD-like immunophenotypic profile, whereas GS patients that display T-cell CM and myDC defects in blood would suffer from more severe infections, even under IgRT.

In summary, our results show that except for a deeper B-cell defect, the pattern of immune cell alterations found in blood of GS patients is indistinguishable from CVID, whenever conventional serum antibody levels and lymphoid cell analyses based on the recommendation of the current international guidelines for PID screening and classification are followed (30, 31, 73). In contrast, more extended and detailed analyses of the blood lymphoid and innate cell compartments as those performed here, revealed additional cellular defects that involve memory TCD4^+^ and TCD8^+^ cell and myDC populations, typically found among patients with a CID-like GS disorder, associated with a delayed emergence of hypogammaglobulinemia after the onset of the thymoma and a higher rate of opportunistic infections after IgRT had been established. In contrast, patients with concurrent diagnosis of thymoma and hypogammaglobulinemia displayed an altered immune cell profile in blood which is closer to that of B-negative CVID patients, in association with a better response to IgRT. Altogether, these results support the use of more in depth standardized immunophenotypic studies that include the analysis of CM/TM TCD4^+^ and TCD8^+^ cells and CD141^+^ myDC in blood, in patients that present with thymoma and clinical suspicion of immunodeficiency, even when serum Ig levels are normal. However, due to the limited number of GS patients studied here and the yet unpredictable evolution of immunodeficiency after thymectomy in these patients, further confirmatory studies in larger series of GS patients with a longer follow-up are needed to confirm our data.

## Data availability statement

The raw data supporting the conclusions of this article will be made available by the authors, without undue reservation.

## Ethics statement

The studies involving humans were approved by the local Ethics Committees of the participating centers. The studies were conducted in accordance with the local legislation and institutional requirements. The participants provided their written informed consent to participate in this study.

## Author contributions

AT-V: Conceptualization, Data curation, Formal analysis, Investigation, Methodology, Software, Validation, Visualization, Writing – original draft, Writing – review & editing. LA: Resources, Writing – review & editing. SS: Writing – review & editing, Resources. CS: Resources, Writing – review & editing. MM: Resources, Writing – review & editing. JM: Resources, Writing – review & editing. CB: Resources, Writing – review & editing. PA-C: Resources, Writing – review & editing. DC: Resources, Writing – review & editing. PO: Resources, Writing – review & editing. JN: Resources, Writing – review & editing. MH: Resources, Writing – review & editing. SA: Resources, Writing – review & editing. MJ: Writing – review & editing. CP: Visualization, Writing – review & editing. AS: Resources, Writing – review & editing. ÁP: Resources, Writing – review & editing. JD: Writing – review & editing. MP-A: Conceptualization, Data curation, Formal analysis, Funding acquisition, Investigation, Methodology, Project administration, Resources, Software, Supervision, Validation, Writing – original draft, Writing – review & editing. AO: Conceptualization, Funding acquisition, Investigation, Project administration, Supervision, Writing – review & editing, Writing – original draft.
