# Supplementary material for: In-depth blood immune profiling of Good syndrome patients
Source: Front Immunol. 2023 Nov 15;14:1285088. doi: 10.3389/fimmu.2023.1285088 (PMC10684950; doi:10.3389/fimmu.2023.1285088)
Supplement: Supplementary file 1 [file DataSheet_1.pdf]

SUPPLEMENTARY MATERIAL

SUPPLEMENTARY TABLES

Supplementary Table 1. Fluorochrome-conjugated antibody combinations used for flow cytometric identification of subsets of TCD4$^+$ cells, cytotoxic T and NK cells and both monocytes and dendritic cells in blood.

A. EuroFlow® PID Screening tube (PIDOT).

Fluorochrome	BV421	BV510	FITC	PerCP-Cy5.5	PE	PE-Cy7	APC	APC-C750
**Marker**	CD27	CD45RA	CD8 + smIgD	CD4 + smIgM	CD16 + CD56	CD19 + TCRγδ	CD3	CD45
**Clone**	M-T271	HI100	UCHT-4 + IADB6	RPA-T4 + MHM-88	3G8 + C5.9	SA287 + 11F2	UCHT-1	HI30
**Source**	Cytognos							

APC, allophycocyanine; APC-C750, allophycocyanine-C750; BV, brilliant violet; FITC, fluorescein isothiocyanate; PE, phycoerythrin; PECy7, phycoerythrin-cyanine7; PerCPCy5.5, peridinin chlorophyll protein-cyanine5.5; sm, surface membrane

B. EuroFlow® 14-color Mo/DC IMM tube used for the identification of different populations of monocytes and dendritic cells in blood.

Fluoro-chrome	BV421	BV510	BBV605	BV650	BV711	BV786	BB515	PerCP-Cy5.5	PE	PE-CF594	PE-Cy7	APC	AF700	APC- H7
**Marker**	CD141	CD5	CD192	CD62L	HLA-DR	CD16	CD1c	CD36	Slan + FcεRL	CD34	CD33	CD300e + CD303	CD45	CD14
**Clone**	1A4	UCHT2	K036C2	DREG56	G46-6	3G3	F10/21A3	CLB-IVC7	DD-1; AER-37	581	P67.6	UP-H2; AC144	HI30	MϕP9
**Source**	BD	BD	BL	BL	BD	BD	BD	Immunostep	Miltenyi Biotech; Immunostep	BD	BD	Immunostep; Miltenyi Biotech	BD	BD

AF, alexa fluor; APC, allophycocyanine; APC-H7, allophycocyanine-hilite®7; BD, Becton/Dickinson Biosciences; BL, Biolegend; BV, brilliant violet; BB, BD Horizon Brilliant™ Blue family of dyes; DC, dendritic cells; FITC, fluorescein isothiocyanate; IMM, immune monitoring; Mo, monocytes; PE, phycoerythrin; PE-CF594, phycoerythrin-carboxyfluorescein594; PE-Cy7, phycoerythrin-cyanine7; PerCPCy5.5, peridinin chlorophyll protein-cyanine5.5; sm, surface membrane.

C. EuroFlow® 14-color TCD4 IMM tube used for the identification of different populations of TCD4$^+$ cells in blood.

Fluoro-chrome	BV421	BV510	BV650	BV711	BV786	VioBright FITC	PerCP-Cy5.5	PE	PE-CF594	PE-Cy7	APC	AF700	APC-H7
**Marker**	CD27	CD45RA	CD62L	CD127	CD3	CD25	CCR10	CD183	CD196	CD194	CD185	CD45	CD4
**Clone**	M-T271	HI100	DREG56	HIL7R M21	SK7	4E3	1B5	1C6/C XCR3	11A9	L291H4	REA103	HI30	SK3
**Source**	BD	BD	BL	BD	BD	Miltenyi Biotech	BD	BD	BD	BL	Miltenyi Biotech	BD	BD

AF, alexa fluor; APC, allophycocyanine; APC-H7, allophycocyanine-hilite®7; BD, Becton/Dickinson Biosciences; BL, Biolegend; BV, brilliant violet; FITC, fluorescein isothiocyanate; IMM, immune monitoring; PE, phycoerythrin; PE-CF594, phycoerythrin-carboxyfluorescein594; PE-Cy7, phycoerythrin-cyanine7; PerCPCy5.5, peridinin chlorophyll protein-cyanine5.5; sm, surface membrane.

D. EuroFlow® 14-color T/NK cytotoxic IMM tube used for the identification of different populations of cytotoxic T and NK cells in blood.

Fluoro-chrome	BV421	BV510	BV650	BV711	BV786	FITC	PerCP-Cy5.5	PE	PE-CF594	PE-Cy7	AF700	APC-Vio770
**Marker**	CD27	CD45RA	CD62L	CD16	CD3	CD57	CD28	cyGranzyme B	CD8	TCRγδ	CD45	CD56
**Clone**	M-T271	HI100	DREG56	3G8	SK7	HNK1	CD28.2	QA16A02	RPAT8	11F2	HI30	REA196
**Source**	BD	BD	BL	BD	BD	BD	BL	BL	BD	BD	BD	Miltenyi Biotech

AF, alexa fluor; APC, allophycocyanine; BD, Becton/Dickinson Biosciences; BL, Biolegend; BV, brilliant violet; cy, cytoplasmic; FITC, fluorescein isothiocyanate; IMM, immune monitoring; PE, phycoerythrin; PE-CF594, phycoerythrin-carboxyfluorescein594; PE-Cy7, phycoerythrin-cyanine7; PerCPCy5.5, peridinin chlorophyll protein-cyanine5.5; sm, surface membrane.

E. EuroFlow® 12-color immunoglobulin isotype B-cell IMM tube used for the identification of different populations of B-cells in blood.

**Fluoro-chrome**	**BV421**	**BV510**	**BV650**	**BV711**	**BV786**	**FITC**	**PerCP-Cy5.5**	**PE**	**PE-CF594**	**PE-Cy7**	**APC**	**APCH7**
**Marker**	CD27	smIgM	CD24	CD21	CD19	smIgG3 + smIgG2	smIgA1 + smIgA2	smIgG1 + smIgG2	smIgD	CD5	smIgG4 + smIgA1	CD38
**Clone**	M-T271	MHM-88	ML5	B-ly4	SJ25C1	SAG3/ SAG2	SAA1/ SAA2	SAG1/ SAG2	IA6-2	L17F12	SAG4/ SAA1	HB7
**Source**	BD	BL	BD	BD	BD	Cytognos	Cytognos	Cytognos	BD	BD	Cytognos	BD

APC, allophycocyanine; BD, Becton/Dickinson Biosciences; BL, Biolegend; BV, brilliant violet; FITC, fluorescein isothiocyanate; IMM, immune monitoring; PE, phycoerythrin; PE-CF594, phycoerythrin-carboxyfluorescein594; PE-Cy7, phycoerythrin-cyanine7; PerCPCy5.5, peridinin chlorophyll protein-cyanine5.5; sm, surface membrane.

Supplementary Table 2. Clinical manifestations of Good Syndrome patients stratified by the time between the diagnosis of thymoma and the onset of hypogammaglobulinemia (n=9).

	**Simultaneous (n=4)**		**Asynchronous (n=5)**	
	**At diagnosis**	**After Ig replacement therapy**	**At diagnosis**	**After Ig replacement therapy**
**Respiratory tract infections**	100%	50%	80%	100%
**Non-respiratory tract infections**	100%	25%	60%	40%
**Encapsulated bacterial infections**	25%	25%	60%	60%
**Opportunistic infections[a]**	100%	**25%**	60%	**100%***
**Severity of infections[b]**	-	25%	-	80%
**Autoimmunity**	25%	0%	20%	20%
**Lymphoproliferation[c]**	0%	25%	0%	20%
**Enteropathy**	0%	0%	20%	40%
**Neoplastic complications**	0%	25%	0%	20%

Results expressed as percentage of cases. CID, combined immunodeficiency; [a]Opportunistic infections: opportunistic bacterial (eg *Pseudomonas aeruginosa, Pneumocystis jirovecci, Escherichia coli, Campylobacter, Helicobacter pylori*), viral (eg CMV, RSV, SARSCov2) and fungal infections (eg *Candida, Aspergillus*). [b]Severity of infections (>2 hospital admissions due to severe infections); [c]Lymphoproliferation (lymphadenopathy or other organomegalies). *p-value ≤ 0.05 simultaneous *vs.* asynchronous diagnosis of thymoma and hypogammaglobulinemia after Ig replacement therapy.

Supplementary Table 3. Immunophenotypic profile of the 102 populations of main leukocytes and lymphocytes populations, both monocytes and dendritic cells, CD4^{+} T-cells, cytotoxic T and NK cells and B-cells identified in blood employing the EuroFlow$^{®}$ tubes.

**Populations**	**Immunophenotype**
Lymphocytes	FSC^{lo} SSC^{lo} CD45^{hi}
B-cells	CD19^{+} CD3^{-} CD45RA^{+}
immature/transitional	CD45^{hi} CD19^{+} CD27^{-} CD5^{het} CD38^{hi} CD24^{hi} IgD^{+} IgM^{++}
Naïve	CD45^{hi} CD19^{+} CD27^{-} CD5^{het} CD38^{-} CD24^{het} IgD^{++} IgM^{+}
Unswitched memory B-cells (MBC)	CD45^{hi} CD19^{+} CD27^{+} CD5^{-} CD38^{lo} IgM^{++} IgD^{+} IgG1^{-} IgG2^{-} IgG3^{-} IgG4^{-} IgA1^{-} IgA2^{-}
IgG MBC	CD45^{hi} CD19^{+} CD27$^{-/+}$ CD5^{-} CD38^{lo} IgM^{-} IgD^{-} IgG^{+} IgA^{-}
IgA MBC	CD45^{hi} CD19^{+} CD27$^{-/+}$ CD5^{-} CD38^{lo} IgM^{-} IgD^{-} IgG^{+} IgA^{+}
Plasma cells (PC)	SSC^{int} CD19^{lo} CD27^{+} CD5^{-} CD38^{hi} CD21^{-} CD24^{-}
IgM^{+} PC	IgM^{+} IgD^{-} IgG^{-} IgA^{-}
IgG^{+} PC	IgM^{-} IgD^{-} IgG^{+} IgA^{-}
IgA^{+} PC	IgM^{-} IgD^{-} IgG^{-} IgA^{+}
T-cells	CD3^{+} CD19^{-} CD16^{-}CD56$^{-/lo}$
TCRαβ^{+} T-cells	TCRγδ^{-}
CD4^{+} T-cells	CD4^{+} CD8^{-}
Naïve CD4^{+} T-cells	CD3^{+} CD4^{+} CD25^{-} CD127^{+} CD183^{-} CD185^{-} CD194^{-} CD196^{-} CCR10^{-} CD27^{+} CD45RA^{+} CD62L^{+}
CM CD4^{+} T-cells	CD3^{+} CD4^{+} CD25$^{lo/+}$ CD127^{+} CD183$^{-/+}$ CD185$^{-/+}$ CD194$^{-/+}$ CD196$^{-/+}$ CCR10$^{-/+}$ CD27^{+} CD45RA^{-} CD62L^{+}
TM CD4^{+} T-cells	CD3^{+} CD4^{+} CD25$^{lo/+}$ CD127^{+} CD183$^{-/+}$ CD185$^{-/+}$ CD194$^{-/+}$ CD196$^{-/+}$ CCR10$^{-/+}$ CD27^{+} CD45RA^{-} CD62L^{-}
EM CD4^{+} T-cells	CD3^{+} CD4^{+} CD25^{-} CD127$^{-/+}$ CD183$^{-/+}$ CD185$^{-/+}$ CD194$^{-/+}$ CD196$^{-/+}$ CCR10$^{-/+}$ CD27^{-} CD45RA^{-} CD62L$^{-/+}$
TE CD4^{+} T-cells	CD3^{+} CD4^{+} CD25^{-} CD127$^{-/+}$ CD183$^{-/+}$ CD185$^{-/+}$ CD194$^{-/+}$ CD196$^{-/+}$ CCR10$^{-/+}$ CD27^{-} CD45RA^{+} CD62L$^{-/+}$
TFH	CD3^{+} CD4^{+} CD25$^{-/lo}$ CD127$^{-/+}$ CD183$^{-/+}$ CD185^{+} CD194$^{-/+}$ CD196$^{-/+}$ CCR10^{-}
CM TFH	CD27^{+} CD45RA^{-} CD62L^{+}
TM TFH	CD27^{+} CD45RA^{-} CD62L^{-}
EM TFH	CD27^{-} CD45RA^{-} CD62L$^{-/+}$
TE TFH	CD27^{-} CD45RA^{+} CD62L$^{-/+}$
Treg	CD3^{+} CD4^{+} CD25^{hi} CD127^{lo} CD183$^{-/+}$ CD185$^{-/+}$ CD194$^{-/+}$ CD196$^{-/+}$ CCR10$^{-/+}$
CM Treg	CD27^{+} CD45RA^{-} CD62L^{+}
TM Treg	CD27^{+} CD45RA^{-} CD62L^{-}
EM Treg	CD27^{-} CD45RA^{-} CD62L$^{-/+}$
TE Treg	CD27^{-} CD45RA^{+} CD62L$^{-/+}$
Th1	CD3^{+} CD4^{+} CD25$^{-/+}$ CD127$^{-/+}$ CD183^{+} CD185^{-} CD194^{-} CD196^{-} CCR10^{-}
CM Th1	CD27^{+} CD45RA^{-} CD62L^{+}
TM Th1	CD27^{+} CD45RA^{-} CD62L^{-}
EM Th1	CD27^{-} CD45RA^{-} CD62L$^{-/+}$
TE Th1	CD27^{-} CD45RA^{+} CD62L$^{-/+}$
Th2	CD3^{+} CD4^{+} CD25$^{lo/+}$ CD127$^{-/+}$ CD183^{-} CD185^{-} CD194^{+} CD196^{-} CCR10^{-}
CM Th2	CD27^{+} CD45RA^{-} CD62L^{+}
TM Th2	CD27^{+} CD45RA^{-} CD62L^{-}
EM Th2	CD27^{-} CD45RA^{-} CD62L$^{-/+}$
TE Th2	CD27^{-} CD45RA^{+} CD62L$^{-/+}$
Th17	CD3^{+} CD4^{+} CD25$^{lo/+}$ CD127$^{-/+}$ CD183^{-} CD185^{-} CD194^{+} CD196^{+} CCR10^{-}
CM Th17	CD27^{+} CD45RA^{-} CD62L^{+}
TM Th17	CD27^{+} CD45RA^{-} CD62L^{-}
EM Th17	CD27^{-} CD45RA^{-} CD62L$^{-/+}$
TE Th17	CD27^{-} CD45RA^{+} CD62L$^{-/+}$
Th22	CD3^{+} CD4^{+} CD25$^{lo/+}$ CD127$^{-/+}$ CD183^{-} CD185^{-} CD194^{+} CD196^{+} CCR10^{+}
CM Th22	CD27^{+} CD45RA^{-} CD62L^{+}
TM Th22	CD27^{+} CD45RA^{-} CD62L^{-}
EM Th22	CD27^{-} CD45RA^{-} CD62L$^{-/+}$
TE Th22	CD27^{-} CD45RA^{+} CD62L$^{-/+}$
Th1/Th17	CD3^{+} CD4^{+} CD25$^{lo/+}$ CD127$^{-/+}$ CD183^{+} CD185^{-} CD194^{-} CD196^{+} CCR10^{-}
CM Th1/Th17	CD27^{+} CD45RA^{-} CD62L^{+}
TM Th1/Th17	CD27^{+} CD45RA^{-} CD62L^{-}
EM Th1/Th17	CD27^{-} CD45RA^{-} CD62L$^{-/+}$

TE Th1/Th17	CD27^{-} CD45RA^{+} CD62L$^{-/+}$
Th1/Th2	CD3^{+} CD4^{+} CD25$^{lo/+}$ CD127$^{-/+}$ CD183^{+} CD185^{-} CD194^{+} CD196$^{-/+}$ CCR10$^{-/+}$
CM Th1/Th2	CD27^{+} CD45RA^{-} CD62L^{+}
TM Th1/Th2	CD27^{+} CD45RA^{-} CD62L^{-}
EM Th1/Th2	CD27^{-} CD45RA^{-} CD62L$^{-/+}$
TE Th1/Th2	CD27^{-} CD45RA^{+} CD62L$^{-/+}$
CD8^{+} T-cells	CD4^{-} CD8^{+}
Naïve CD8^{+} T-cells	CD3^{+} CD8^{+} TCRγδ^{-} CD27^{+} CD28$^{+/++}$ CD45RA^{+} CD62L^{+}
CM CD8^{+} T-cells	CD3^{+} CD8^{+} TCRγδ^{-} CD27^{+} CD28$^{+/++}$ CD45RA^{-} CD62L^{+}
TM CD8^{+} T-cells	CD3^{+} CD8^{+} TCRγδ^{-} CD27^{+} CD28$^{-/++}$ CD45RA^{-} CD62L^{-}
EM CD8^{+} T-cells	CD3^{+} CD8^{+} TCRγδ^{-} CD27^{-} CD28$^{-/++}$ CD45RA^{-} CD62L$^{-/+}$
EE CD8^{+} T-cells	CD3^{+} CD8^{+} TCRγδ^{-} CD27^{lo} CD28^{-} CD45RA^{+} CD62L$^{-/+}$
TE CD8^{+} T-cells	CD3^{+} CD8^{+} TCRγδ^{-} CD27^{-} CD28^{-} CD45RA^{+} CD62L$^{-/+}$
DNT T-cells	CD4^{-} CD8$^{-/lo}$
Naïve DNT T-cells	CD27^{+} CD45RA^{+}
CM/TM DNT T-cells	CD27^{+} CD45RA^{-}
EM DNT T-cells	CD27^{-} CD45RA^{-}
EE DNT T-cells	CD27^{lo} CD45RA^{+}
TE DNT T-cells	CD27^{-} CD45RA^{+}
TCRγδ^{+} T-cells	TCRγδ+
Naïve TCRγδ^{+} T-cells	CD3^{+} CD8^{-} TCRγδ^{+} CD27^{+} CD28$^{+/++}$ CD45RA^{+} CD62L^{+}
CM TCRγδ^{+} T-cells	CD3^{+} CD8^{-} TCRγδ^{+} CD27^{+} CD28$^{+/++}$ CD45RA^{-} CD62L^{+}
TM TCRγδ^{+} T-cells	CD3^{+} CD8^{-} TCRγδ^{+} CD27^{+} CD28$^{+/++}$ CD45RA^{-} CD62L^{+}
EM TCRγδ^{+} T-cells	CD3^{+} CD8^{-} TCRγδ^{+} CD27^{-} CD28$^{-/++}$ CD45RA^{-} CD62L$^{-/+}$
EE TCRγδ^{+} T-cells	CD3^{+} CD8^{-} TCRγδ^{+} CD27^{lo} CD28^{-} CD45RA^{+} CD62L$^{-/+}$
TE TCRγδ^{+} T-cells	CD3^{+} CD8^{-} TCRγδ^{+} CD27^{-} CD28^{-} CD45RA^{+} CD62L$^{-/+}$
NK-cells	CD19^{-} CD3^{-} CD16^{+}CD56^{hi} CD45RA$^{lo/+}$
CD56^{lo} NK-cells	CD3^{-} CD16^{+} CD45RA$^{lo/+}$ CD27$^{-/+}$ CD56^{lo}
CD56^{hi} NK-cells	CD3^{-} CD16^{+} CD56^{hi} CD45RA$^{lo/+}$ CD27$^{-/+}$
Monocytes	FSC^{int} SSC^{int} CD45$^{+/hi}$ CD19^{-} CD3^{-} CD14$^{-/+}$CD16^{-}CD56$^{-/+}$
Classical monocytes	CD14^{+} CD16^{-} CD33^{++} CD36^{++} CD141^{lo} CD192^{+} CD300e^{lo} CD303^{-} HLA-DR$^{+/++}$
Intermediate monocytes	CD14^{+} CD16^{+} CD33^{++} CD36^{+} CD141^{+} CD192^{+} CD300e^{+} CD303^{-} HLA-DR^{++}
Non-classical monocytes	CD14$^{-/lo}$ CD16^{+} CD33$^{+/++}$ CD36$^{-/+}$ CD141^{+} CD192$^{-/+}$ CD300e^{++} CD303^{-} HLA-DR^{+}
Neutrophils	FSC^{hi} SSC^{hi} CD45^{hi} CD19^{-} CD3^{-} CD16^{hi}CD56$^{-/+}$
Basophils	FSC^{int} SSC^{int} CD45^{lo} CD19^{-} CD3^{-} CD16^{-} CD56^{-} CD45RA^{-} CD27^{-}
Eosinophils*	FSC^{hi} SSC^{hi} CD45^{hi} CD19^{-} CD3^{-} CD16^{-} CD56^{-}
Dendritic cells (DC)	FSC^{int} SSC^{int} CD45^{+} CD14$^{-/lo}$ CD16^{-} CD33$^{-/++}$ HLA-DR^{++} Slan^{-}
CD1c^{+} myeloid DC	CD1c$^{lo/+}$ CD5$^{-/+}$ CD14$^{-/lo}$ CD16^{-} CD33^{++} CD36^{+} CD141$^{lo/+}$CD300e$^{-/+}$ CD303^{-}
CD141^{+} myeloid DC	CD1c^{-} CD5$^{-/lo}$ CD14^{-} CD16^{-} CD33^{++} CD36^{-*} CD141^{++} CD300e^{-} CD303-
Plasmacytoid DC	CD1c^{-} CD5^{-} CD14^{-} CD16^{-} CD33^{-} CD36^{+} CD141^{+} CD300e^{-} CD303^{+}

FSC, forward scatter; SSC, side scatter; lo, low expression; hi, high expression; MBC, memory B-cells; TCR, T-cell receptor; NK, natural killer; DNT, double negative T-cells (CD4-CD8- TCRγδ-); CM, central memory; TM, transitional memory; EM, effector memory; EE, early effector; TE, terminal effector; TFH: follicular helper T cell; Th: T helper; Tregs: regulatory T cells; cy, cytoplasmic. *Cell population displays autofluorescence in the channel employed for the study of the maker.

SUPPLEMENTARY FIGURES

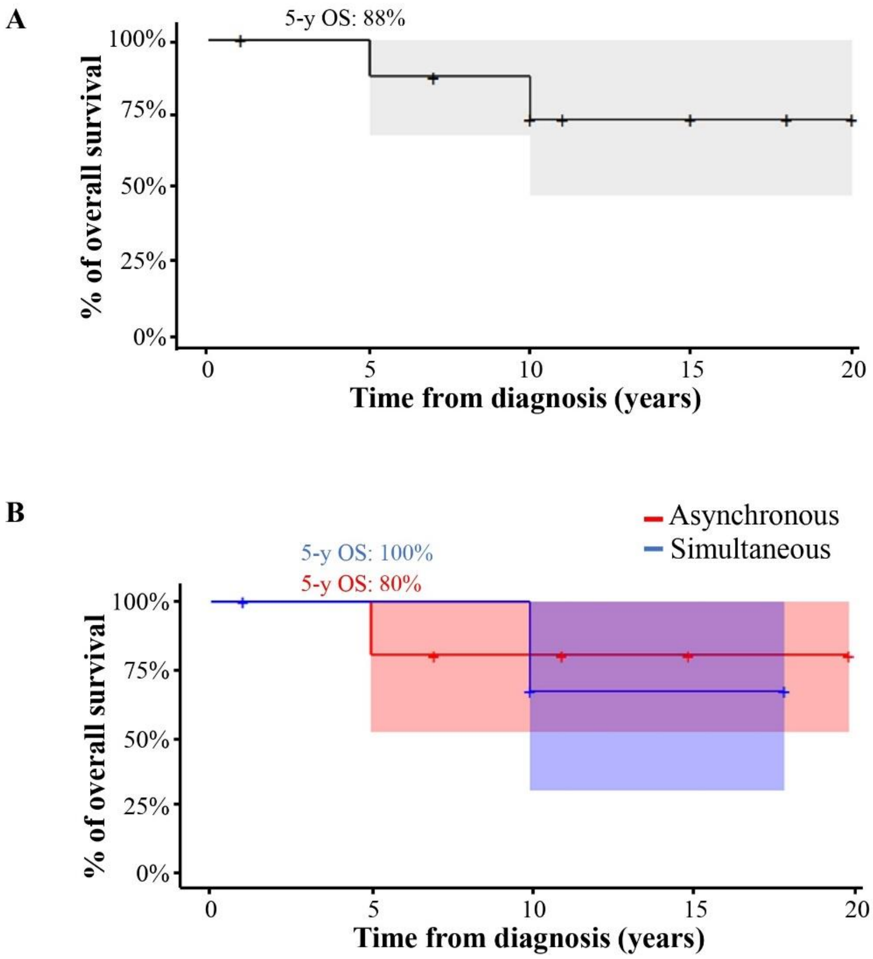

Supplementary Figure 1. Overall survival of GS patients (panel A) stratified according to the time between the diagnosis of thymoma and the onset of hypogammaglobulinemia (panel B). Blue line represents OS curve from patients with simultaneous thymoma and hypogammaglobulinemia diagnosis and red line from patients with asynchronous diagnosis; shadow areas indicated the 95% CI of each curve; + individual cases. GS, Good syndrome; OS, overall survival; CI, confidence interval; y, years.

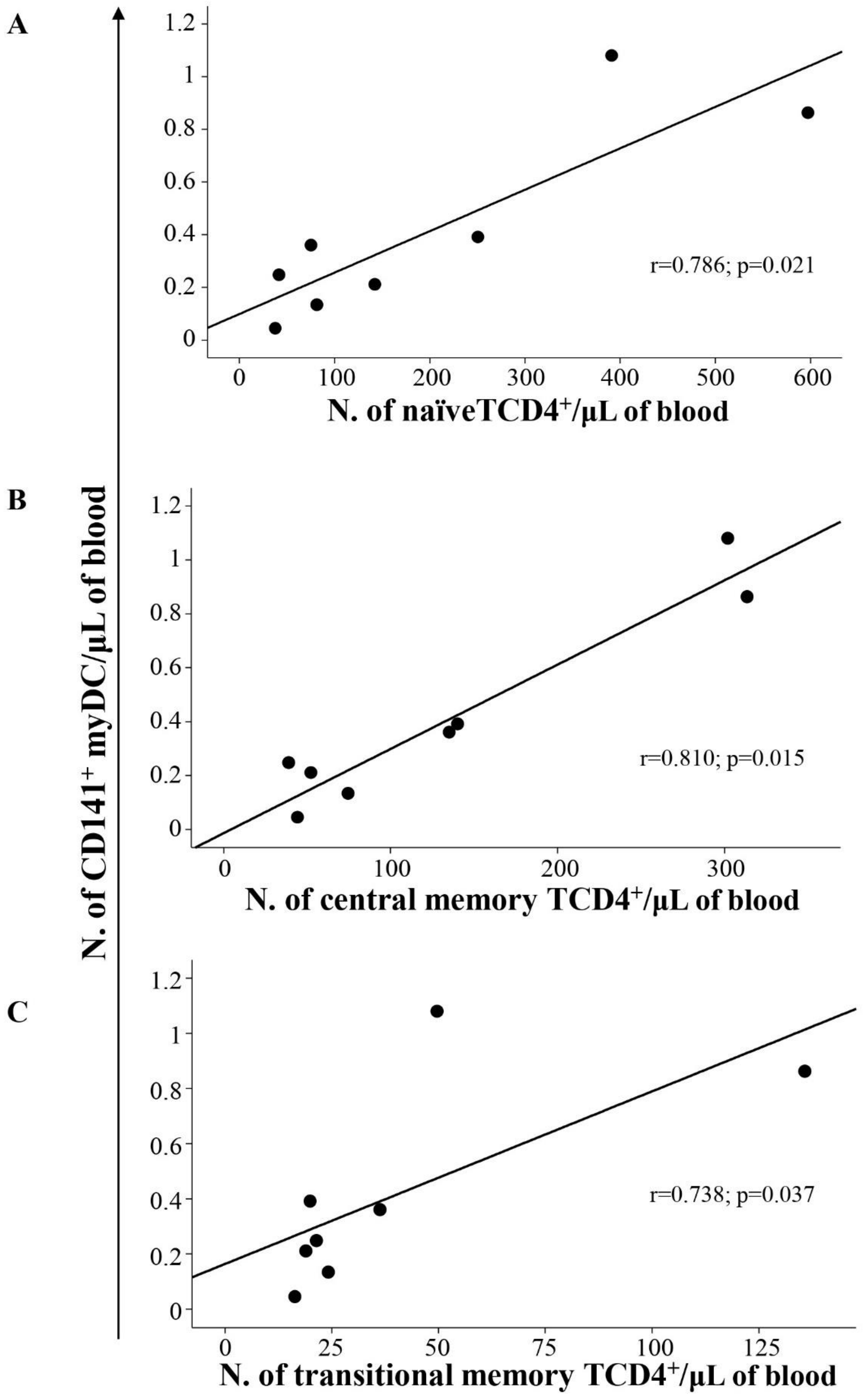

Supplementary Figure 2. Correlation between CD141⁺ myDC and both the TCD4⁺ naïve (panel A), central memory (panel B) and transitional memory (panel C) cell counts. Dark points represent each GS patient and the dark line represents the trend line fitted to the data points. r: Spearman correlation coefficient; myDC: myeloid dendritic cells.

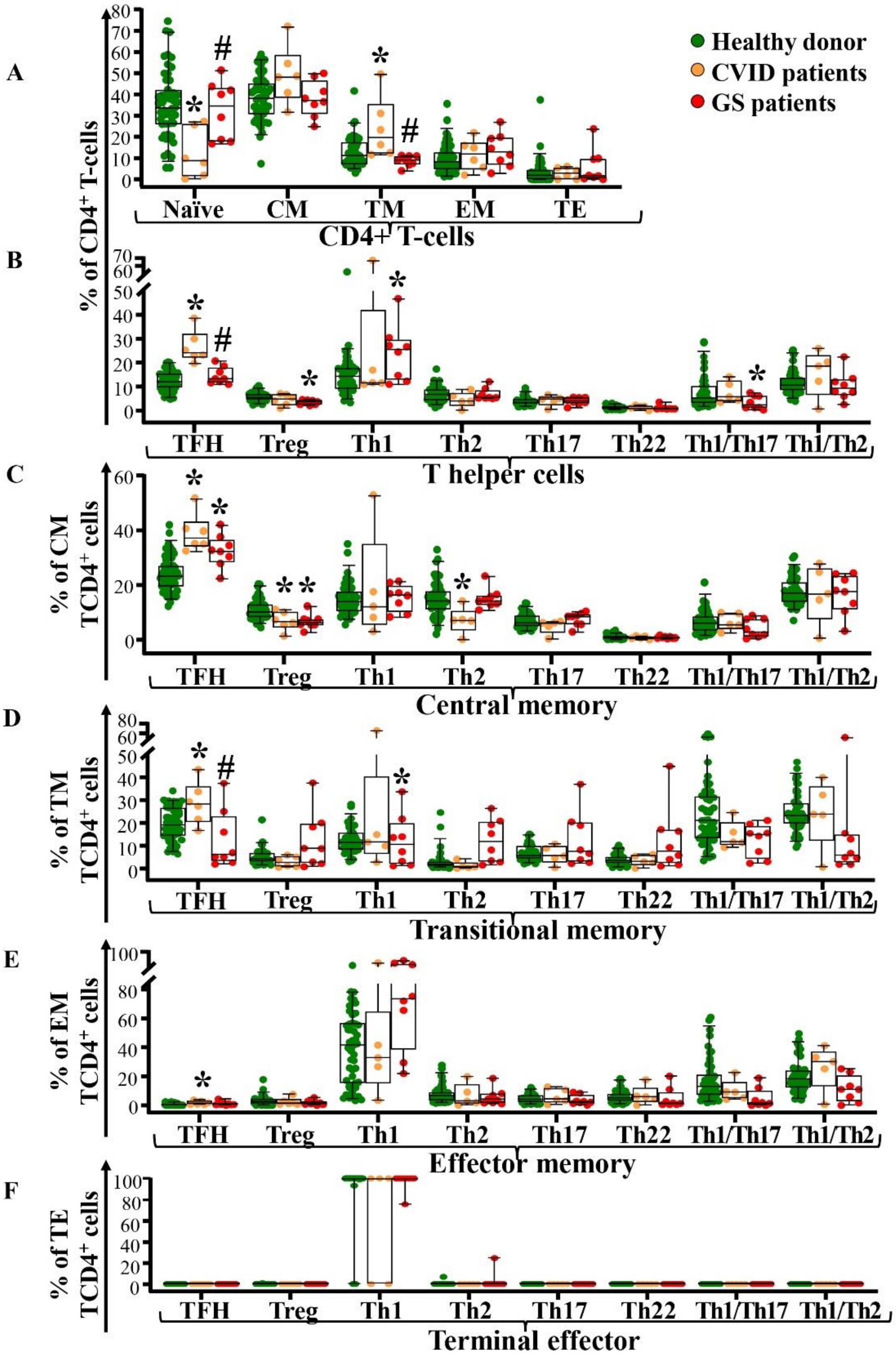

Supplementary Figure 3. Relative frequency of maturation-associated subsets and functional populations of CD4+ T-cells in blood of GS patients (n=8) compared to age-matched CVID patients (n=6) and healthy donors (n=51). Relative frequency of maturation-associated subsets of CD4+ T-cells (naïve, central memory -CM-, transitional memory -TM-, effector memory -EM-, and terminal effector -TE- cells) (panel A) and functional subsets (TFH, Treg, Th1, Th2, Th17, Th22, Th1/Th17 and Th1/Th2) (panel B) of central memory CD4+ T-cells (panel C), transitional memory CD4+ T-cells (panel D), effector memory CD4+ T-cells (panel E) and terminal effector CD4+ T-cells (panel F) in blood of GS patients vs. age-matched healthy donors, are shown. Notched boxes extend from the 25th to the 75th percentile values, while the line in the middle and vertical lines correspond to median values and the 5th and 95th percentiles, respectively; individual cases are represented as green dots (healthy donors), orange dots (CVID) and red dots (GS). *p-value ≤ 0.05 *vs.* healthy donors. #p-value ≤ 0.05 *vs.* CVID.

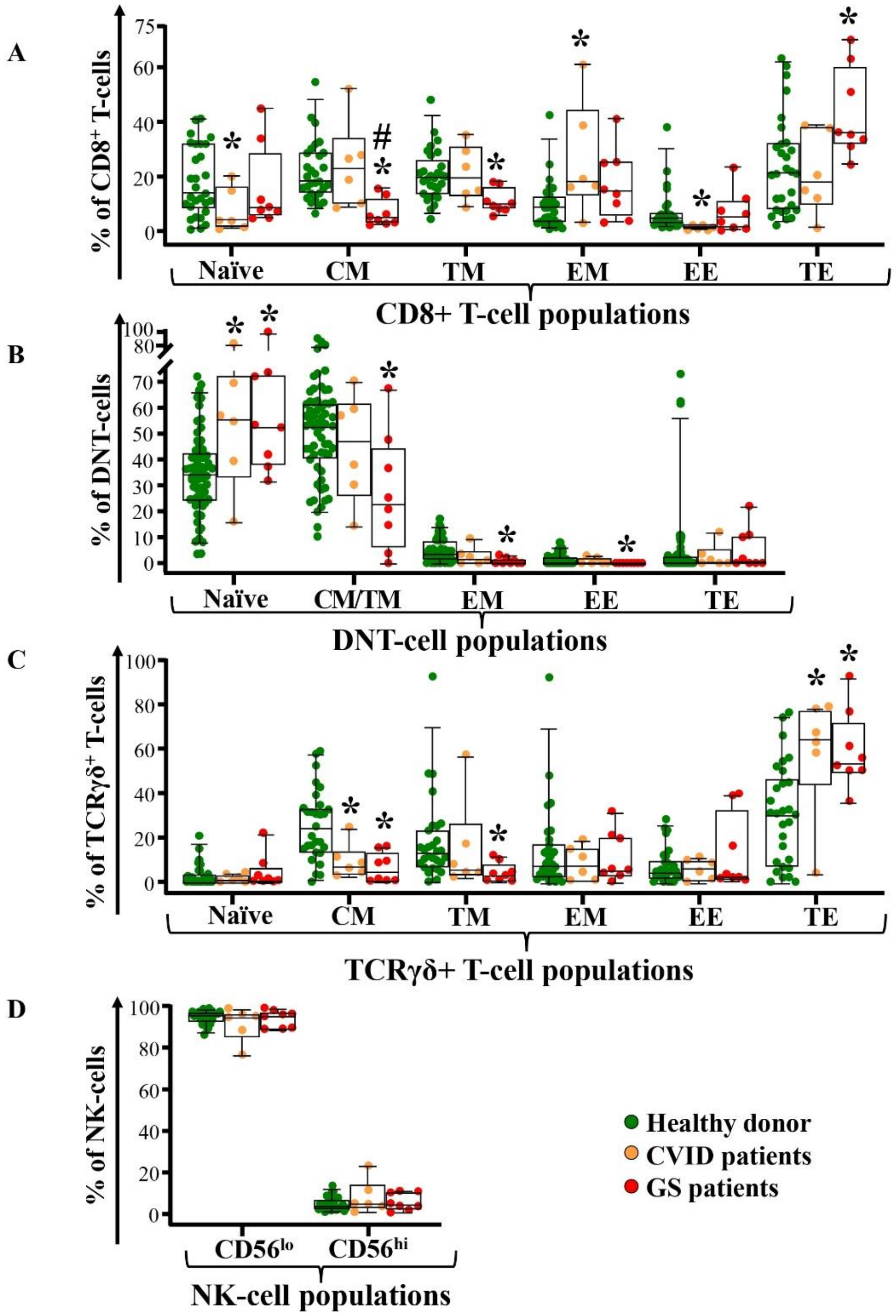

Supplementary Figure 4. Relative frequency of different maturation-associated populations of cytotoxic T-cells and NK-cells in blood of GS patients (n=8) compared to age-matched matched CVID patients (n=6) and healthy donors (n=51). Relative frequency of different subsets of blood CD8+ T-cells, DN-TCRαβ+ T-cells, and TCRγδ+ T-cells defined according to their maturation stage (naïve, central memory -CM-, transitional memory -TM-, effector memory -EM-, early effector -EE-, and terminal effector -TE-) are shown in panels A, B and C, respectively. In panel D, NK-cell subsets defined by the levels of CD56 expression in peripheral blood from patients with GS and age-matched healthy donors. Notched boxes extend from the 25th to the 75th percentile values, whereas the line in the middle and vertical lines correspond to median values and the 5th and 95th percentiles, respectively; individual cases are represented as green dots (healthy donors), orange dots (CVID) and red dots (GS). CVID, common variable immunodeficiency; GS, Good syndrome; n, number of cases; DNT, double negative T-cells (CD4-CD8- TCRγδ-); TCR, T-cell receptor; NK, natural killer. *p-value ≤ 0.05 *vs.* healthy donors. #p-value ≤ 0.05 *vs.* CVID.

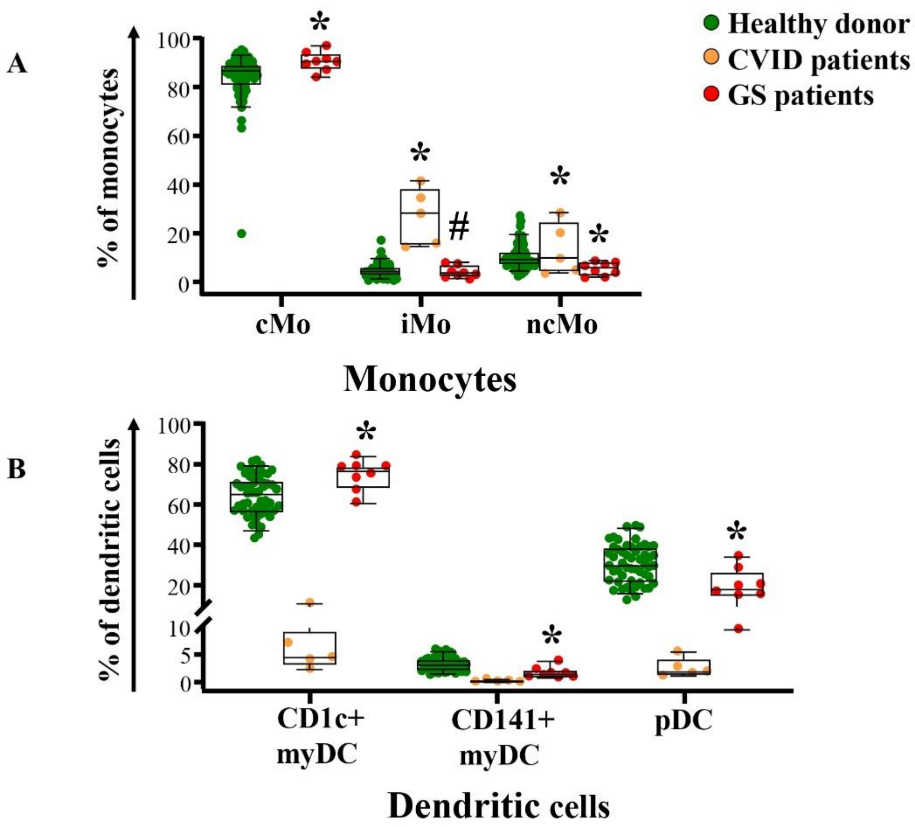

Supplementary Figure 5. Relative frequency of different populations of monocytes and dendritic cells in blood of GS patients (n=8) compared to age-matched matched CVID patients (n=6) and healthy donors (n=51). Relative frequency of the major subsets of monocytes (cMo, iMo and ncMo) (panel A) and both total DC and their subsets of total myDC, CD1c+ myDC, CD141+ myDC and pDC (panel B) in blood of GS patients vs. age-matched healthy donors, are shown. Notched boxes extend from the 25th to the 75th percentile values, while the line in the middle and vertical lines correspond to median values and the 5th and 95th percentiles, respectively; individual cases are represented as green dots (healthy donors), orange dots (CVID) and red dots (GS). CVID, common variable immunodeficiency; GS, Good syndrome; n, number of cases; Mo, monocytes; DC, dendritic cells; cMo, classical monocytes; iMo, intermediate monocytes; ncMo, non-classical monocytes; myDC, myeloid dendritic cells; pDC, plasmacytoid dendritic cells. *p-value ≤ 0.05 *vs.* healthy donors. #p-value ≤ 0.05 *vs.* CVID.
